# Identification of high-yielding and stable Egyptian soybean genotypes for breeding across varied environments

**DOI:** 10.1186/s12870-025-07942-4

**Published:** 2026-01-09

**Authors:** F. E. Waly, A. A. Abou Zied, KH. A. Mourad, Mohamed Abdelghany

**Affiliations:** 1 Food Legume Research Department, Agriculture Research Center, Field Crops Research Institute, Giza, Egypt; 2 Oil Crops Dept. Field Crops Research Institute, Agriculture Research Center, Giza, Egypt; 3 Crop Science Department, Faculty of Agriculture, Damanhour University, Damanhour, 22516 Egypt

**Keywords:** Drought stress, Multi-trait stability index, Yield stability, Genotype × environment interaction

## Abstract

This study evaluated the phenotypic stability of five Egyptian soybean genotypes (Giza 111, Giza 22, and lines 105, 127, and 129) across six environments comprising three water regimes of irrigation levels of 100% (normal), 75% (moderate drought), and 50% (severe drought) of field capacity, evaluated over two growing seasons (2023 and 2024). Morpho-developmental traits (germination percentage, days to flowering and maturity, plant height, hair number, defoliation percentage), yield components (branch number, seed size, pod number, empty pod number, 100-seed weight, seed yield per plant and feddan), and seed biochemical composition (protein and oil contents) were assessed. Morphological characterization revealed that Giza 22 and Line 101 exhibited superior germination rates, while Line 129 demonstrated maximum plant height and number of hairs. Giza 22 showed the earliest maturity date. For yield component analysis, Lines 127 and 129 achieved the highest seed yield, while Giza 22 displayed superior seed size and 100-seed weight, whereas Line 105 exhibited the lowest number of empty pods. The seed biochemical analysis demonstrated that Line 129 achieved maximum protein levels, whereas Line 105 attained the highest oil content. Furthermore, multi-trait stability assessment identified Giza 111 and Line 129 as phenotypically stable across multiple parameters, with Line 129 exhibiting optimal performance-stability equilibrium. This was corroborated by the weighted average of absolute scores from BLUPs (WAASB), where Line 129 recorded the lowest WAASB value, indicating superior multi-trait stability. Collectively, these findings suggest that Line 129 is a stable, high-yielding soybean genotype suited to variable conditions, including drought, making it a valuable resource for improving yield and stability in water-limited environments in Egypt. Further research should investigate the molecular and physiological traits behind its performance and the potential of Giza 111 in breeding for drought tolerance.

**Supplementary Information:**

The online version contains supplementary material available at 10.1186/s12870-025-07942-4.

## Introduction

Soybean (*Glycine max* L.) ranks among the world’s most economically and nutritionally important leguminous crops and and is among the important sources of high-quality protein and oil used directly for human consumption [1]. Owing to the growing demand for plant-based protein and the variety of its byproducts, soybean output has increased dramatically worldwide in recent decades [2]. Important producers such as the United States of America, Brazil, and Argentina contribute significantly to global output, and soybean production plays an important role in providing food security and supporting agricultural economies [1]. Soybean oil is a highly valued soybean seed product and accounts for a large proportion of the vegetable oil produced worldwide [3]. It is valued for its nutritional value since it is rich in polyunsaturated fatty acids, particularly linoleic acid, and its role in ensuring cardiovascular health [4]. In addition, soybean oil is a food industry ingredient with multiple uses in cooking, baking and as a base for salad dressings and margarine. In addition to its use in food applications, soybean oil is also industrially utilized, such as in the production of biodiesel, lubricants, and other cosmetic products, further increasing its economic importance. Because it contains all nine essential amino acids needed for human consumption, soybean protein is a complete protein and of very high nutritional quality [5]. It is included in many processed foods, including dairy and meat substitutes, as well as protein supplements, to meet the increasing demand for plant-based foods. Additionally, its use in animal feed greatly improves the productivity and nutrition of livestock, which contributes to the global food chain [6].

Soybean production is of particular importance in Egypt for improving agricultural sustainability and reducing Egypt’s dependence on imported protein sources [7]. Its integration into cropping systems, especially rotation with cereals, has considerable benefits, including the enhancement of soil fertility through nitrogen fixation and pest management [8]. The yield of soybeans in Egypt remains relatively low due to environmental constraints and suboptimal production practices [9]. Egypt’s soybean is grown for various purposes, including ensuring food security and industrial uses [10]. The primary uses of crops are the generation of soybean oil and meal, which are constituents of local diets and animal feed, respectively [11]. Additional efforts to grow soybeans in Egypt have focused on practicing improved farming practices, using high-yielding and stress-tolerant genotypes, and efficient irrigation management to address environmental concerns [11]. Research projects and policy interventions are designed to promote soybean cultivation, considering its potential to reduce the country’s reliance on imported legumes and oils and promote sustainable agricultural growth [12]. The expansion of soybean fields is a national priority, which necessitates the breeding of cultivars that are tolerant to widespread abiotic stresses such as drought [12].

Drought is one of the most devastating abiotic stresses affecting crop production worldwide [13]. Its impacts on plants are holistic and involve various aspects of plant growth and development [14]. Morphologically, drought tends to lead to decreases in plant height, leaf area, and root biomass, all of which are essential for optimal photosynthesis and nutrient uptake [15]. Physiologically, drought stress leads to water scarcity in plant tissues, followed by a decline in the efficiency of photosynthesis, stomatal closure, and alterations in transpiration rates [15]. These physiological disruptions can further impair a plant’s ability to continue metabolic activities, thereby reducing its growth potential [16]. Drought stress also adversely affects yield-specific traits, such as the number of pods, seed size, and seed weight, eventually leading to reduced crop yields [5]. Drought also significantly impacts seed composition, including oil and protein contents, both of which are important determinants of soybean economic value and nutritional quality [17]. Under normal conditions, soybean seeds contain approximately 18–20% oil and 35–40% protein, but these values significantly vary under water deficit conditions [18]. Drought stress imposed during the reproductive and seed-filling stages can disrupt assimilate partitioning and enzymatic processes underlying lipid and protein biosynthesis and lead to alterations in seed composition [1]. Understanding the physiological and biochemical responses of soybean genotypes to drought is very important during cultivar development for ensuring consistent seed quality under stress conditions [13].

In Egypt, the drought challenge is further exacerbated because Egypt relies on the Nile River for irrigation, which is becoming more unpredictable because of the effects of climate change and geopolitics [19]. Water flow in the Nile has come under pressure because of dam construction in upstream countries, which has taken a toll on the water supply for Egyptian agriculture [20]. Furthermore, Egypt’s temperature and low rainfall throughout the year pose another risk to drought stress, particularly in regions outside of the Nile Delta [20]. Agriculture, which is strongly dependent on irrigation, is at significant risk of water scarcity, imposing danger on crop yields and food security [21]. The increasing population increases the demand for drought-resistant varieties of crops, such as drought-resistant soybean genotypes, which also grow [22]. Research on increasing drought tolerance in crops can help alleviate these issues and promote sustainable agricultural production in Egypt, which is a valuable sector of the economy [21].

The evaluation of the stability and adaptability of soybean cultivars across diverse environmental conditions is pivotal for the recognition of drought-tolerant genotypes [23]. Stability analysis, via the use of statistical models such as estimated marginal means (EMMs) and Genotype plus genotype-by-environment interaction (GGE) biplots, provides a comprehensive overview of genotype × environment interactions and enables the recognition of cultivars with stable performance under both stress and non-stress conditions [24]. The drought tolerances of soybean varieties grown in Egypt differ, so their stability needs to be evaluated under contrasting water regimes [7]. Stability analysis allows breeders to identify genotypes with stable performance under heterogeneous environmental conditions, including drought-stress environments [24]. Genotype × environment interactions under water stress conditions are crucial for the selection of cultivars with high yield potential as well as stable drought tolerance [23]. The goals of this study were to (1) evaluate the mean performance and the stability of the five soybean genotypes in multiple environments, with a particular focus on the selection of genotypes that are both very productive and exhibit stable performance under varying environmental conditions; and (2) determine the adaptation potential of some Egyptian genotypes for potential use in soybean improvement programs and large-scale production based on their adaptation and stability levels.

## Materials and methods

### Plant materials

A field experiment assessed the drought tolerance of specific genotypes of Egyptian soybeans under various water regimes. Five genotypes of soybeans from the Field Crop Research Department, Agriculture Research Centre, Egypt, were used in the study, including Giza 22, Giza 111, Line 105, Line 127, and Line 129 (Table 1). These genotypes were selected based on their previously reported good responses to drought stress, and high yield and oil productivity under Egyptian field conditions [25, 26]. Their tolerance to water deficits characterizes Giza 111 and Line 129, while the Giza 127 cultivar has a high yield. Giza 22 thrives in the hot climate of Upper Egypt, and the Line 105 has a high oil yield.

**Table 1:** Origin and pedigree of the assessed genotypes of soybeans

Genotypes	Pedigree	Country of origin	Pubescence density	Maturity group
Giza 111	Crawford x Celest	Egypt	Heavy	IV
Giza 22	Crawford x Forrst	Egypt	Light	IV
Line 127	D89-8940 x Giza 83	Egypt	Light	V
Line 129	Giza 35 x Giza 83	Egypt	Heavy	V
Line 105	Giza35 x Lamar	Egypt	Heavy	V

In addition to their phenotypic characteristics, the genotypes used in this study are the result of targeted breeding programs conducted at the Field Crops Research Institute. Giza 111 and Giza 22 are advanced cultivars derived from crosses involving elite local and international parental lines (e.g., Crawford × Celest and Crawford × Forrest, respectively), while Lines 105, 127, and 129 are advanced breeding lines developed from multiple generations of selection for drought tolerance, yield potential, and seed quality. These lines represent the latest outputs from Egypt’s national soybean improvement program. Detailed pedigree and genotypic information, including molecular marker profiles and selection history, are available upon request. To support research transparency and reproducibility, all five soybean genotypes evaluated in this study are maintained in the soybean germplasm collection of the Field Crops Research Institute (ARC, Egypt) and are available to researchers upon reasonable request through the corresponding author.

The experiment involved a randomized complete block design (RCBD) with three replicates of each genotype per environment. Each plot measured 3 × 4 m, comprising three rows of each genotype. The row space was 50 cm, and the plants were planted 10 cm apart within rows to ensure optimal plant density and minimize competition.

### Experimental environments and water regime treatments

This study was conducted at the Itay El-Baroud Research Station’s field experimental farm in El-Beheira Governorate, Egypt (Table S1). The experiments were conducted across six environments comprising three water regimes applied over two consecutive growing seasons (2023 and 2024). The three irrigation levels tested were: 100% field capacity (normal irrigation), 75% field capacity (moderate drought stress), and 50% field capacity (severe drought stress). This combination of three water regimes × two seasons resulted in six distinct environments: Env1 (2023, normal irrigation), Env2 (2023, moderate drought), Env3 (2023, severe drought), Env4 (2024, normal irrigation), Env5 (2024, moderate drought), and Env6 (2024, severe drought). These conditions were designed to simulate a sequence of water availability scenarios to evaluate Egyptian soybean genotypes’ performance, yield potential, and stability under varying degrees of water stress. The soil at the experimental site was identified as clay loam, with a pH of [7.5] (Table S2). All the treatments received the same standard agronomic treatments, including weed control and fertilizer. Each experimental plot had three rows for each genotype and was measured [3 × 4 m]. The distance between seeds was 10 cm between plants and 50 cm between rows. Throughout the trial, standard agronomic procedures, such as fertilization, weed control, and insect management, were used. Irrigation was stopped for 7 days during the reproductive stage to induce drought stress. Three methods of irrigation were used: control (well-watered, WW): Throughout the trial, the plants were watered to 100% field capacity. Plants under moderate drought stress (MD) were given [75%] of their field capacity. The plants under severe drought stress (SD) experienced a field capacity of [50%]. The Field capacity was monitored using the gravimetric method and maintained by irrigating when moisture dropped below 80% of field capacity.

### Morphological and developmental measurements

- Germination percentage: Germination was tested under field conditions. Germinated seeds were counted 7 days after sowing per plot, and the following formula was used to calculate the percentage of germination: germination percentage = (number of germinated seeds/total seeds sown) ×100 [27].
- Days to flowering (DH, days): Days from sowing to the day when 50% of the plants in each plot opened the first flower were recorded [28].
- Days to maturity (DM, days): Determined when 90% of the plants were at the full pod maturity stage, with yellow leaves and fully mature seeds [29].
- Plant height (PH, cm): At the physiologically mature stage, 10 random plants per plot were taken from ground level to the end of the main stem on a measuring scale. The average height was recorded [30].
- Binocular field count, or the number of hairs (NH): Five randomly chosen plants in each plot had their leaves sampled. The number of trichomes (hairs) was counted using a stereo microscope at 40× magnification on the adaxial surface of the middle leaflet of the third fully expanded trifoliate leaf from the tip. Counts averaged per cm² area with a micrometric grid [31].
- Percent defoliation: At the mid-pod development stage, defoliation was visually evaluated by calculating the percentage of leaves lost as a result of drought stress [32]. Plants were scored using a five-point scale: 0% (no defoliation), 25% (slight defoliation), 50% (moderate defoliation), 75% (severe defoliation), and 100% (complete leaf loss).

### Yield-related traits

For yield characteristics, data were collected from ten plants per plot at physiological maturity. Pod number, empty pods, seed size, and seed yield per plant were counted manually. The 100-seed weight was taken using a precision electronic balance after seeds were fully dried. Seed yield per hectare (SY/ha, kg/ha) was estimated from plot yield and adjusted to standard moisture content. The number of branches (NB): Ten randomly chosen plants per plot were counted for the number of primary branches at the flowering stage [33]. Seed size (SZ, mm³): A graduated cylinder was used to measure the water displacement of one hundred seeds from each genotype. The unit of measurement for volume was cubic millimeters (mm³) [34]. Pods’ number for each plant (NP/P): Ten plants from each plot were randomly selected at harvest, and the number of pods on each plant was tallied [35].

The number of empty pods (NEP): From the same ten randomly selected plants, the number of empty pods devoid of seeds was counted and recorded separately [35]. 100-seed weight (HSW, g): 100 seeds for each genotype were weighed via an electronic balance, so that the seeds were well dried before weighing [36]. Seed yield per plant (SY/P, g): The total weight of 10 randomly selected plants per plot was weighed via an electronic balance and averaged [37]. Seed yield per hectare (SY/ha, kg/ha): Seed yield was calculated on a plot basis and extrapolated to kilograms per hectare (1 feddan ≈ 0.42 ha). Seed yield per hectare = total plot yield (kg)/plot area (m²) × 10,000 [37].

### Seed chemical components

The protein content (%) was determined via the Kjeldahl method [38]. The nitrogen content of ground seed samples of each genotype was analyzed, and the percentage protein content was estimated following the conversion factor shown in the equation below:

Protein% = total nitrogen content×6.25.

Oil content (%) was extracted using Soxhlet extraction method [39]. After six hours of extracting a known weight of powdered seed material via petroleum ether, the oil percentage was determined by dividing the extracted oil weight by the sample weight, then multiplied by100.

### Germplasm availability

All genotypes are maintained in the soybean germplasm collection at the Field Crops Research Institute (ARC, Egypt) and are available upon reasonable request from the corresponding author or directly from the Field Crops Research Institute germplasm bank.

### Statistical analysis

The experiment was treated as a split-plot RCBD in which irrigation regime (main plot factor) was assigned to the main plots and genotypes (subplot factor) were assigned within each main plot. A homogeneity test was performed across all traits using Bartlett’s test. All the statistical analyses were conducted via R (version 4.4.3). GGE biplots were used to graphically display the genotype‒environment interactions. To analyze the mean performance of the genotypes and compare them in terms of their stability, the mean performance vs. stability method was employed. The analyses were conducted at the α = 0.05 significance level, and the results were interpreted with corresponding p values and visual plots. The Multi-Trait Stability Index (MTSI) Analysis was computed to identify genotypes that also possess combined high mean performance and stability for most traits. Calculation of the MTSI was done via the use of the WAASB method implemented through the ‘metan’ package in R (version 4.4.3). The intensity of selection of 15% was employed to choose superior genotypes.

Using the genotype plus genotype-by-environment interaction (GGE) biplot model, Additive Main Effects and Multiplicative Interaction (AMMI) analysis, Estimated Marginal Means (EMMs) analysis and mean performance vs. stability, the genotype‒environment interaction (GEI) was evaluated. The following model was used to perform GGE biplot analysis in [R 4.4.3 software]:

$$\mathrm{Yij}-\mu-\beta\mathrm{j}=\lambda1\alpha_\mathrm{i}1\gamma\mathrm{j}1+\lambda2\alpha_\mathrm{i}2\gamma\mathrm{j}2+\epsilon_\mathrm{ij}$$

where µ is the grand mean, Yij is the mean performance of genotype i in environment j, βj​ is the environmental effect, λ1​ and λ2 are the singular values, α_i1_​ and α_i2_ are the genotype scores, γj1​ and γj2 are the environment scores, and ε_ij_​ is the residual error.

## Results

### The analysis of variance (ANOVA) for all studied traits

The Analysis of variance (ANOVA) indicated that genotype (G) exerted highly significant effect (*p* < 0.01) on all the characters except germination percentage where it was the sole significant factor, suggesting strong genetic control (Table S3). Environment also showed significant influence on most of the characters, particularly days to maturity, plant height, defoliation percentage, yield traits, protein, and oil content. While, characters such as germination and pods per plant were not greatly affected by environment. G × E interactions were noted for several characters such as days to maturity, plant height, defoliation, seed size, empty pods, yield traits, protein, and oil, indicating that the genotypes responded differently in various environments. Meanwhile, days to flowering, percent germination, hairs per plant, and pods per plant manifested non-significant G × E interactions (Table S3).

### Analysis of the morphological traits

AMMI analysis revealed that ENV, GEN, and GEN × ENV influenced all the morphological traits, except germination percentage (Table S4). The effect of genotype alone was extremely significant for germination, which implies high genetic control with minimal environmental or interaction influence. For days to flowering, genotype and environment had significant effects, but genotype × environment interaction was not significant (Table S4). Genotype, environment, and their interaction had highly significant effects on days to maturity, plant height, and defoliation (Table S3). The number of hair characteristics was also affected and significantly differed among them because of the influence of genotype and environment. However, the genotype × environment interaction was not significant, indicating genotypic stability in terms of performance (Table S4).

### Analysis of yield-related and chemical characteristics

For branch number, genotype, environment, and their interaction, all had significant effects, indicating strong genetic and environmental influences (Table S5). Similarly, seed volume was highly significantly affected by genotype, environment, and their interaction, indicating a multiplicative interaction between these factors. On the other hand, the number of pods had a very strong genotype effect, and the environmental and interaction effects were not statistically significant, revealing the predominant role of genetic factors in this trait (Table S4). Genotype, environment, and their interaction effects were found to make significant contributions to the number of empty pods, revealing the sensitivity of this trait to intrinsic as well as extrinsic factors. For 100-seed weight, both genotypic and environmental variation existed, but there was no significant G×E interaction, which indicates that the performance of the genotype was rather uniform across environments (Table S5). Both the seed yield per plant and the seed yield per hectare had genotypic, environmental, and interaction high effects, which indicate the complexity and variability of the yield components under different environmental conditions (Table S4). According to the AMMI analysis of variance, protein and oil contents were found to be significantly impacted by genotype, environment, and their interactions, as shown in Table S6.

### EMMs analysis of morphological, yield-related traits, and seed chemical components

Variation between the five soybean genotypes was observed for all the parameters measured in all the environments. For germination percentage, Line 105 recorded the highest in environments 1 and 2 (95.7%), while the lowest was observed in Giza 111 under environment 4 (79.3%) (Table S7). For days to flower, the most was shown by Line 129 in environment 2 (49.7 days), and the least by Giza 22 in environment 6 (38.3 days) (Table S6). The longest days to maturity were also shown by Line 129 in environments 1 and 4 (136 days), while the shortest maturity duration was shown by Giza 22 in environments 3 and 6 (114 days) (Table S6). Concerning the height of plants, Line 129 possessed the tallest in environment 4 (121.7 cm), whereas the shortest were Line 127 and Giza 22 in environment 3 (71.7 cm and 65.0 cm) (Table S6). Maximum hair density was in Line 129 in environment 3 (27.33), whereas Line 127 possessed minimum hair density in environments 1 and 4 (6.67) (Table S7). Finally, in percentage defoliation, Giza 22 had the highest in environment 3 (32.33%), while Line 105 had the lowest in environments 1 and 4 (0.0%) (Table S7).

The highest branch values were recorded in Giza 111, particularly in environments 3 and 6 (3.00 and 2.86, respectively), indicating increased branching capacity. Line 127 indicated the lowest branch values in all the environments, with a reading of between 0.06 (Env2) and 1.66 (Env3) (Table S8). Giza 22 possessed the largest seed size index among environments, with the largest value recorded in Env1 (56.8) and consistent performance in others. Line 129 possessed the smallest seed size values, varying from 24.5 in Env6 to 29.0 in Env4 (Table S8). The values for the number of pods per plant were maximum for Line 105 (48.4 in Env2 and 48.3 in Env3) and Line 129 (50.8 in Env5), which indicated maximum pod-yielding potential. Minimum NP/P values were recorded in Giza 22, though in Env5 and Env6 more prominently (26.0 and 25.6, respectively) (Table S8). The maximum number of empty pods was recorded in Giza 111 and Line 127, i.e., in Env3 (12.54 and 12.48, respectively) and Env6 (11.66 and 11.8, respectively). The minimum empty pods were recorded in Giza 111 in Env1 (2.97) (Table S8). Giza 22 maintained the highest seed weight consistently in all the environments, ranging from 14.1 (Env3) to 17.2 (Env1, 2, and 4). Line 105 showed the lowest hundred-seed weight, ranging from 10.6 (Env3) to 12.3 (Env4) (Table S8).

The highest seed yield per plant was in Line 127, with values at maximum in Env1 and Env4 (18.2 and 17.7, respectively). The values were minimum in Line 105, particularly in Env3 and Env6 (11.0 and 11.4, respectively) (Table S8). Line 127 had the highest seed yield per hectare under Env1 and Env4, followed by Giza 22 (Table S8). The lowest SY/ha was in Giza 111, especially under Env6 and Env3 (Table S8).

Based on the marginal means of protein and oil contents across five genotypes of soybeans in six environments, the highest content of protein was quoted under Line 129 in Environment 1 (43.1%), while the lowest content of protein was quoted under Environment 6 in Giza 111 (25.6%) (Table S9). The highest content of oil was quoted under Environment 6 in Line 105 (23.2%), while the lowest content of oil was quoted in Line 129 under Environment 1 (14.4%) (Table S9).

### Correlation analysis

**Fig. 1:**
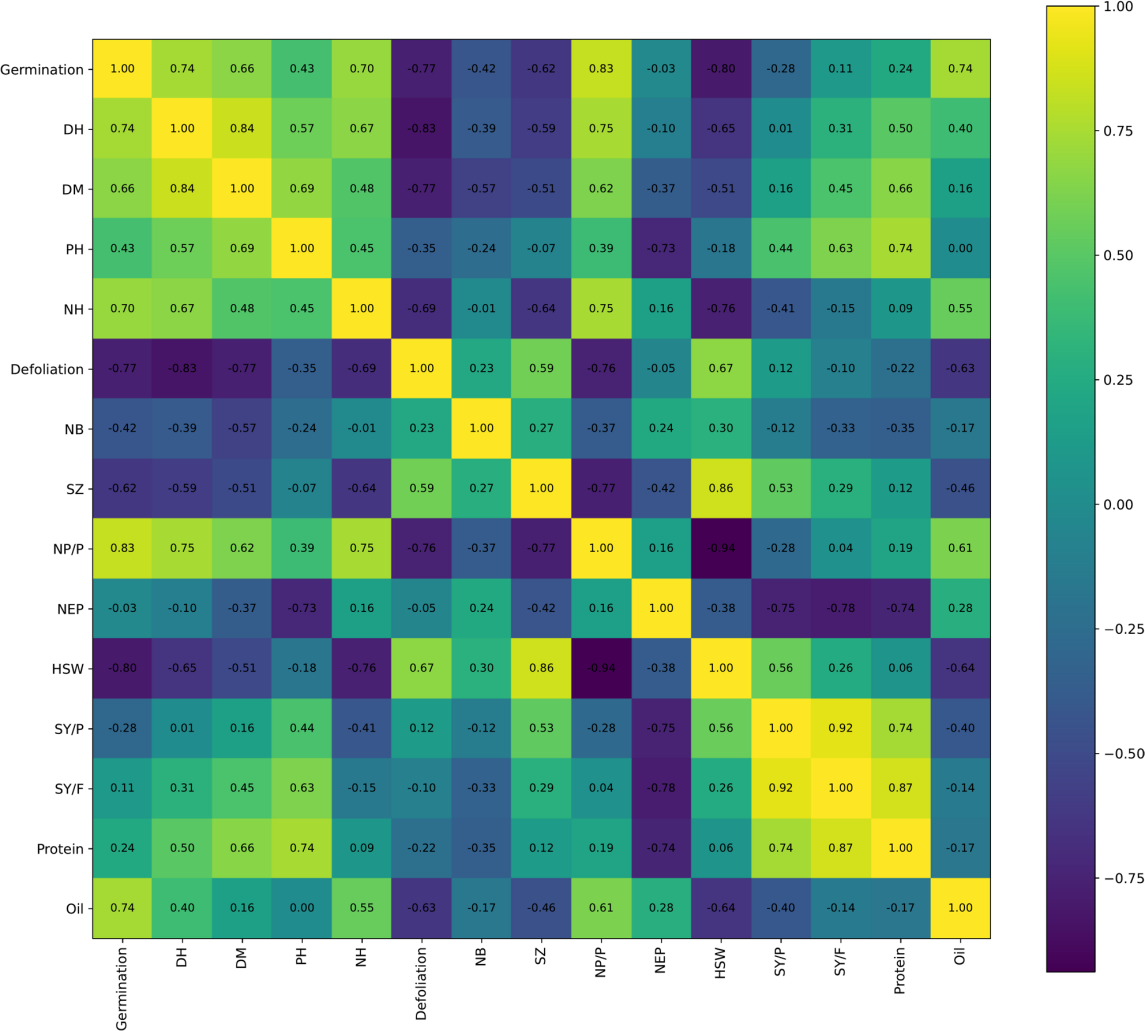
Correlation heatmap of morphological, yield-related and chemical traits in soybean. The green colors indicate positive correlations and the blue colors indicate negative correlations, with color intensity indicating the strength of the correlation. The analysis identifies significant trait interrelationships of interest for soybean yield performance and stress response

For some of the greatest positive correlations (*r* ≥ 0.80), these included those for seed yield per plant (SY/P) and seed yield per hectare (SY/ha) (*r* = 0.92), SY/ha and protein content (*r* = 0.87), seed zone length (SZ) and hundred seed weight (HSW) (*r* = 0.86), and days to heading (DH) with days to maturity (DM) (*r* = 0.84) (Fig. 1). Germination was also highly positively correlated with the number of pods per plant (NP/P) (*r* = 0.83). On the other hand, the highest negative correlation was between NP/P and HSW (*r* = −0.94), which reflects an inverse proportionality of seed size and number of pods. Other notable negative correlations included defoliation with DH (*r* = −0.83), DM (*r* = −0.77), and germination (*r* = −0.77), and germination with HSW (*r* = −0.80) (Fig. 1), indicating the destructive effects of defoliation and seed vigor on plant growth and seed weight. Moderate correlations (0.30 ≤ |r| < 0.80) were in general, such as the positive correlations between germination and DM (*r* = 0.66), oil content (*r* = 0.74), and NH (*r* = 0.70), and between DH and NP/P (*r* = 0.75), and PH with SY/F (*r* = 0.63) and protein (*r* = 0.74) (Fig. 1). SY/P was also moderately and positively correlated with HSW (*r* = 0.56) and protein (*r* = 0.74) (Fig. 1). In contrast, the weakest correlations (|r| ≤ 0.10) were between NEP and germination (*r* = −0.03), SY/P and DH (*r* = 0.01), NB and NH (*r* = −0.01), and oil content with PH (*r* = 0.00) (Fig. 1), indicating weak or zero linear relationships between those pairs. The results are enlightening regarding critical stress interdependencies and trade-offs that have implications for improving soybean productivity and stress tolerance with strategic breeding strategies.

### GGE biplot of morphological characteristics

For germination, the first two principal major components (PC1: 95.54%, PC2: 3.10%) explained 98.64% of total variance (Fig. S1). Environments 5 and 6 possessed small angles between their vectors and thus were strongly positively correlated, also, environments 3 and 4 were strongly correlated. Line 105 was the most suitable genotype for Environments 1 and 4, whereas Line 129 was the most suitable for Environments 5 and 6. Giza 111 was the best cultivar for Environment 3(Fig. S1). For days to Flowering, PC1 and PC2 explained 99.87% of total variation (PC1: 98.91%, PC2: 0.96%) (Fig. S1). There was a positive correlation between all Environments. Most environments clustered in the same direction, indicating a similar effect. Acute angles (Env3 and Env5) indicated a strong positive correlation. There was also a strong correlation between Env2 and both Environments 4 and 5. Environments 1 and 6 also showed a strong correlation between them. Line 129 was suitable in performance for Environments 2 and 4, whereas Line 105 was suitable for Environment 3 (Fig. S1). Related to days to maturity, PC1 (97.45%) and PC2 (2.16%) together contributed 99.61% of the variation (Fig. S1). Env3 and Env6 were in proximate vectors (high correlation), also Env1 and Env2 had a high positive correlation. Line 129 was the best genotype for Environment 5, whereas Line 105 was the best for Environment 4.

In terms of plant height, PC1 and PC2 together explained 97.47% of the variation (PC1: 90.54%, PC2: 6.93%) (Fig. S1). All Environments showed high correlation. The strongest correlation was found between Env 4 and Env 6. Env1 had a strong positive correlation with both Environments 2 and 6 were highly positively correlated with each other. Line 129 was the most suitable genotype for Environments 3 and 4, while Line 105 was the best genotype for Env3 (Fig. S1). Concerning the number of hairs, PC1 and PC2 accounted for 99.92% of total variation (PC1: 99.54%, PC2: 0.38%) (Fig. S1). All six Environments were positively correlated, especially between the second and fourth Environments, the first and third environments, the first and fourth Environments, where the correlation was highly significant. Line 105 performed well for all Environments, whereas Line 129 performed the best for Environment 6. Giza 111 was the best for Environment 5. In terms of defoliation characteristics, the biplot accounted for 99.85% of the total variation (PC1: 98.58%, PC2: 1.27%). All of the environments were positively correlated, with the strongest correlation observed between Env2 and Env3, Env1 and Env4, and Env2 and Env5. Giza 111 and Line 129 presented the highest defoliation in all Environments (Fig. S1).

### GGE biplot, relationship among environments and genotypes for yield-related and chemical characteristics

**Fig. 2:**
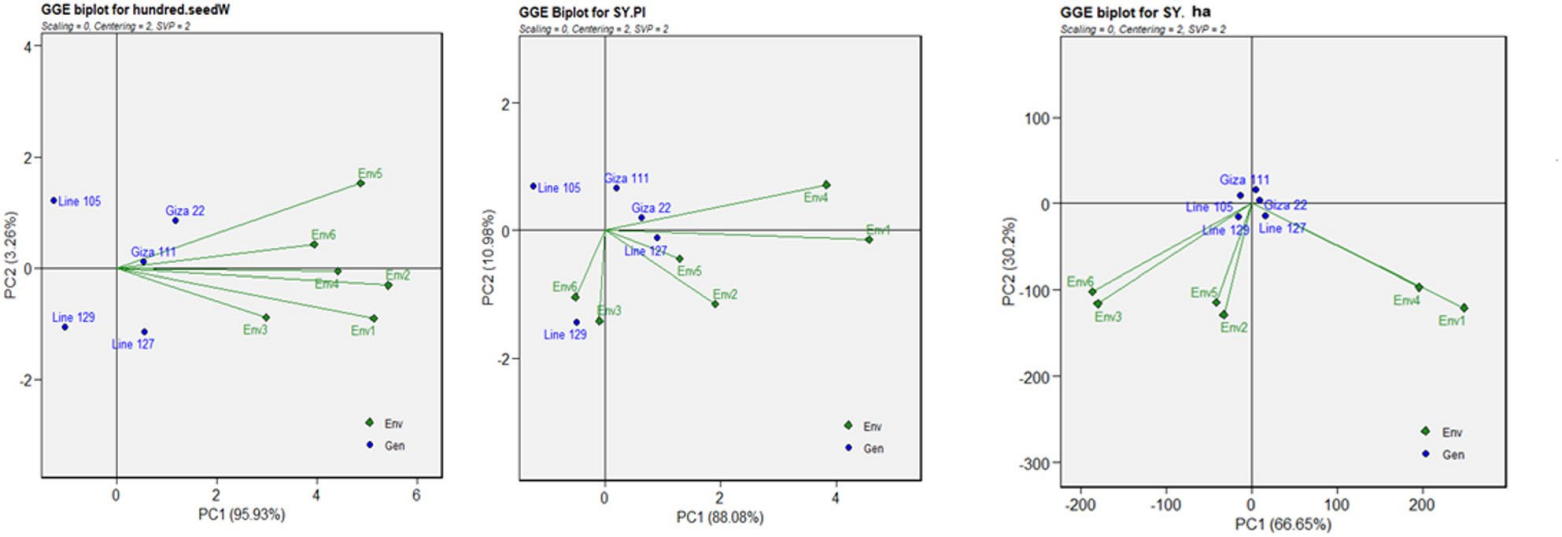
GGE biplot showing relationships among environments (green diamonds) and genotypes (blue circles) for yield-related parameters (100-seed weight (g), seed yield per plant (g) and seed yield per hectare (kg/ha)). Type of biplot: relationship biplot (environment vectors and genotype positions)

Principal component analysis (PCA) of nine yield-related traits showed high variation and large genotype-by-environment (G × E) interaction patterns. PC1 and PC2 accounted for 98.47% variation for the number of branches (PC1: 96%, PC2: 2.47%) (Fig. S2). Env2 and Env5 had high positive correlations. Also, Environments 1 and 4 Giza 111 showed a high positive correlation with each other. Giza 111 was the most adaptable for Environment 3, while Giza 22 was the best for Environments 1, 4 and 5 (Fig. S2). In seed size, 98.67% of the variation was accounted for by the first two principal components (PC1: 86.32%, PC2: 12.35%) (Fig. S2). The highest correlation was found between Environments 3 and 5. Also, Environments 3, 4, and 5 showed a considerable correlation among them. Giza 22 was generally good across environments 3, 5 and 6. For the pods number, PC1 and PC2 explained 99.22% of the variation (PC1: 94.99%, PC2: 4.23%) (Fig. S2). Environments were grouped differently, with Env1 and Env3 showing high positive correlations. Environments 2 and 4 also showed a high positive correlation. Line 05 showed good performance for Environment 3, while Line 129 showed the best adaptability for Environment 5 (Fig. S2). Related to the number of empty pods, PC1 and PC2 accounted for 98.95% of the variation (PC1: 84.59%, PC2: 14.36%). There was a negative correlation between environment 3 and both environments 2, 4 and 5, while there was a positive correlation between environments 3 and 6. Environments 1, 2, 4 and 5 also showed a strong correlation with each other. Giza 111 was the best performing and most suitable for the third environment (Env 3), while Line 127 was the best for the sixth environment (Env 6). Line 129 showed high performance in the environments 1, 2, 4 and 5 (Fig. S2). In terms of hundred-seed weight, PC1 explained 95.93% of the variation. The second and fourth environments had a strong positive correlation, and the fourth and sixth environments also showed a strong correlation (Fig. 2). Giza 22 Giza 111 performed the best in Environments 5 and 6, while Line 127 was well suited to Env3.

Regarding the seed yield per plant, PC1 explained 88.08% of the variation (Fig. 2). All environments showed a significant positive correlation with each other. Env2 and Env5 were highly positively correlated. Also, Env 3 and Env 6 had a highly significant correlation. Giza 22 performed well in Environment 4, while Line 127 performed well in Environment 5 levels. Line 129. Giza 22 performed highly in the fourth environment (Env 4), while Line 127 performed highly in the fifth environment (Env 5). Line 129 performed highly in environments 3 and 6 (Fig. 2). Concerning hectare yield of seeds, the total variance was 96.85% (PC1: 66.65%, PC2: 30.2%). Env3 and Env5 were strongly discriminative and correlated with each other. Also, Environments 2 and 6 had a highly significant correlation. Line 127 had a good performance for Environments 1 and 4(Fig. 2). Line 129 showed an excellent performance for Environments 2, 3, 5 and 6 (Fig. 2).

A significant amount of the overall genotype × environment interaction (GEI) variation was explained by the GGE biplots for protein and oil contents. In terms of protein content, PC1 explained 77.29% of the variation, whereas PC2 explained 20.24%, for a total of 97.53% (Fig. S3). A cumulative 99.89% explanation of the overall fluctuation was provided by the 89.5% contribution of PC1 and the 10.39% contribution of PC2 to the oil content, indicating extremely trustworthy and instructive biplots (Fig. S3). While Environments 3 and 5 were closely linked, suggesting a comparable response pattern, Environments 2 and 4 were highly positively correlated with protein content (Fig. S3). Env1 and Env4, as well as Environments 3 and 6, exhibited strong correlations with the oil content. Also, there was a strong correlation between Environments 2 and 5 for oil content. With respect to the performance of the genotypes, Line 129 was the best in Environment 5 for protein content. Line 105 had the highest amount of protein in Environments 2 and 5 (Fig. S3).

### The mean performance versus the stability of the morphological characteristics

#### Genotype performance

The mean performance vs. stability biplots for all morphological traits varied between the six environments. In germination, Line 105 had the highest average performance, while Line 101 and Giza 22 were the worst (Fig. S4). Giza 22 flowered earliest among the days to flowering, while Line 129 flowered late. For days to maturity, Giza 22 matured the earliest, while Line 129 matured the latest (Fig. S4). Line 129 had the highest plant height, with the lowest being Line 127 (Fig. S4). Lines 127 scored the lowest regarding hair counts, while the best performance belonged to Line 129 (Fig. S4). For defoliation, the highest defoliation was Giza 22. The least defoliated was Line 105 (Fig. S4).

#### Stability assessment

Stability was assessed as the perpendicular distance of each genotype from the average environment coordinate (AEC) abscissa: genotypes closest to the AEC abscissa are interpreted as the most stable across the tested environments. All varieties showed good stability, with the least stable being Line 127 for germination (Fig. S4). In days to flowering, Giza 22 was the most stable. Line 129 had good stability. In terms of days to maturity, Giza 22 was the most stable genotype, while Line 129 was also good in stability. Concerning plant height, Line 127 was the most stable genotype. Line 129 also showed good stability (Fig. S4). Regarding the hair number and defoliation characteristics, the genotypes generally showed relatively small perpendicular distances and therefore comparatively stable (Fig. S4).

### The mean performance vs. stability biplots for the various yield-related and chemical traits

The performance vs. stability biplots for various yield-related and chemical traits revealed distinct differences in both the stability and performance of the five soybean genotypes across the six environments.

#### The genotypes’ mean performance

**Fig. 3:**
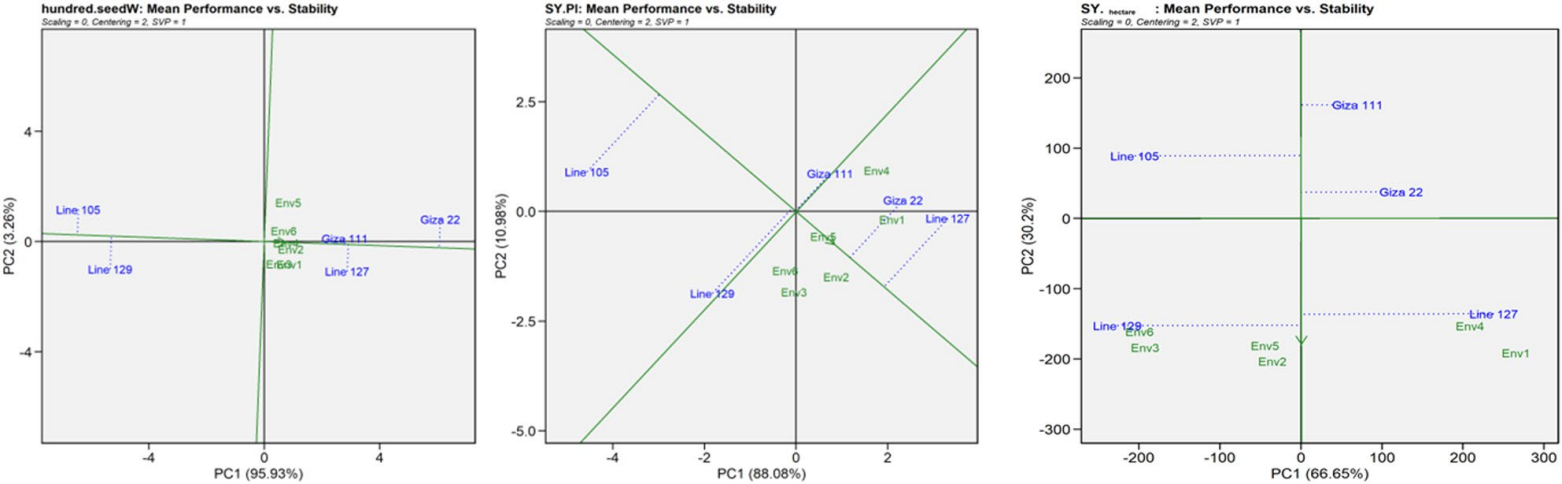
Mean performance compared with the stability biplot showing the yield-related characteristics (100-seed weight (g), seed yield per plant (g) and seed yield per hectare (kg/ha)) of the six settings and five genotypes of soybeans. The first two principal components (PC1 and PC2) are shown in a biplot, with PC1 representing the mean performance and PC2 indicating stability. Genotype performance and stability are determined via average environment coordination (AEC)

For the branches’ number, the highest mean performance was of Giza 111, positioned most on the positive end of the PC1 axis. Line 127 had the lowest mean, positioned most on the negative end (Fig. S5). The environments (Env1 to Env5) were positioned close to the origin, indicating poor interaction effects and uniform conditions among trials. In terms of seed size, Giza 22 showed higher-than-average performance in environments. Line 129 showed lower-than-average performance (Fig. S5). For the pod numbers, Lines 127 and 105 coincided with the performance arrow, with high mean values, while Giza 22 was of low performance. For empty pods, Line 127 contained higher mean values, while Line 105 contained lower values, favorable as a result of fewer empty pods (Fig. S5). Concerning 100-seed weight, Giza 22, which is on the right side of the performance line, possessed better mean performance. Lines 105 and 129, on the left, had lower values (Fig. 2). Related to the seed yield per plant, Line 127 had the highest mean, followed by Giza 22. Line 105 was the poorest with low yields (Fig. 3). Related to the seed yield per hectare, Lines 127 and 129 were the best. Giza 111 was the poorest (Fig. 3).

In protein content, the performance axis was right, where the greatest means are in that direction. This meant that Line 129 was comparatively high in protein content, and the lowest is Giza 111 (Fig. S6). For oil content, the axis of performance also moved right, where Line 105 had relatively higher means. Giza 111, which is distant from the left, possessed the lowest oil content (Fig. S6).

#### Genotype stability

For the number of branches characteristic, all genotypes had high stability, but Line 105 was the least stable genotype (Fig. S5). In terms of seed size, Giza 22 is the most stable genotype because it is nearest to the AEC abscissa (smallest perpendicular distance to the stability axis). Line 127 is the lowest in stability. Concerning the stability of the number of pods, Giza 111, Giza 22 and Line 127 are the most stable genotypes because they seem to be positioned near the performance line (Fig. S5). Line 129 was the least stable genotype as far from the AEC abscissa and indicated a large swing in performance from one environment to another. To the stability of the number of empty pods, Giza 22 was the most stable, as it is closest to the performance line. Line 129 was the least stable (Fig. S5). The 100-seed weight of the Giza 111 genotype was closest to the abscissa of the AEC, indicating that it had the greatest stability in all the environments (Fig. 3). In terms of the stability of the seed yield per plant, Giza 22 and line 129 were the most stable of all the genotypes (Fig. 3). In terms of the seed yield per hectare characteristic stability, Giza 22 and line 129 were the most stable of all the genotypes. In contrast, Line 127 were the least stable (Fig. 3).

For protein content, the most stable protein content genotype was Giza 111 and Line 129, as it is nearest to the AEC line. The least stable line was Line 105 (Fig. S6). In terms of the oil content, Giza 111 and Line 105 were the most stable because it was almost straight on the AEC line. Line 129 was the least stable genotype (Fig. S6).

**Fig. 4:**
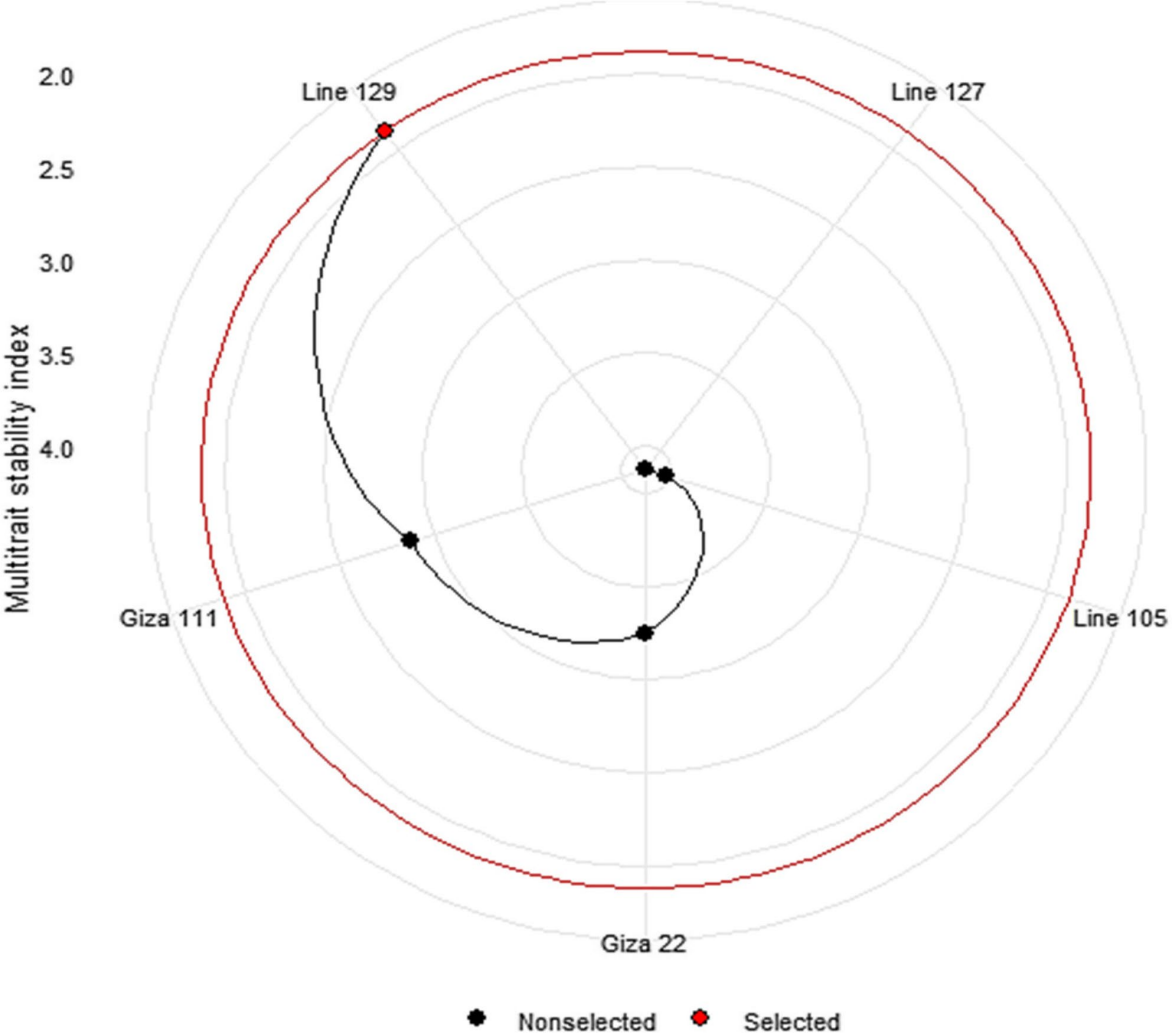
Taking into account six environments and fifteen agronomic features, the soybean genotypes were ranked and chosen according to the multi-trait stability index. Genotypes selected based on a 15% selection intensity are indicated in red, while non-selected genotypes are shown in black

The weighted average of absolute scores from BLUPs (WAASB) based on the multi-trait stability index (MTSI) revealed clear differences in the stability of the five soybean genotypes among the six environments (Fig. 4). The polar plot indicates that ‘Line 129’ possessed the lowest WAASB value, indicating higher multi-trait stability, and hence it was selected (painted in red). In contrast, the other genotypes, Line 127, Line 105, Giza 22, and Giza 111, were located further from the center and were not selected, which means higher WAASB values and lower overall stability (Fig. 4). The ranking ensures that ‘Line 129’ combines good performance with high stability in multiple traits and environments, and it is a suitable option for further breeding and cultivation in fluctuating drought environments.

## Discussion

### AMMI analysis of the studied traits

The AMMI analysis provided important information on the genetic and environmental control of soybean morphological characters. Genotypic effects were highly significant for the majority of the traits, for which genotypic variation explained the majority of the variance, highlighting their breeding value [40]. Environmental effects were significant for all traits except germination, implying that factors like location, temperature, and soil moisture play a significant role in traits like maturity, defoliation, and plant height [40]. Genotype × environment interaction significantly influenced most of the characteristics, which suggested that there were genotypes that performed well in certain environments; therefore, multi-environment testing is important for efficient selection. In contrast, the traits of germination, flowering, hair number, number of pods and 100-seed weight showed non-significant genotype × environment interactions, indicating that there was less variable expression between environments and that selection for these traits may be simple [41].

### EMMs analysis of the studied traits

For days to flowering and maturity, Line 129 consistently took the longest time, suggesting its suitability for long-season environments or delayed flowering strategies [36]. Maximum plant height was recorded in Line 129, particularly in environment 4, reflecting its potential for heavy vegetative growth [42]. Number of hairs, a useful character that may be associated with resistance to insects, was likewise most abundant in Line 129, further suggesting that it may be involved in physical defense mechanisms [43]. Giza 111 displayed superior branching, which may be accountable for vegetative vigor, but did not manifest itself as the most efficient yield [44]. Despite good NEP and NP/P in some environments, its seed yield per plant and feddan were comparatively the lowest, particularly for Env6 and Env3. Line 127 performed exceptionally well as one of the best, with good SY/P and SY/F, especially in Env1 and Env4. It had the lowest branch number but compensated with favorable pod setting and preferred seed yield components. It consistently performed well in several traits and environments. Line 129 performed relatively well in SY/ha and NP/P, especially in Env3 to Env6, because of its flexibility and high-quality pod yield [45]. However, it had a relatively small seed size, which may be a drawback in commercial applications in markets that prioritize large seeds [46]. Line 129 consistently occupied the first position for protein accumulation, especially in Environment 1, implying high genetic potential for protein production under high protein, possibly aided by specific environmental conditions therein [47]. For oil content, the most superior genotype was Line 105, particularly in Environment 6, showing its enhanced capacity for lipid accumulation, possibly attributed to its genetic composition and favorable interaction with the environmental factors in that environment [48]. These results pinpoint Line 127 and Line 129 as the leading challengers for favorable conditions, with Line 127 being exceptional in terms of yield stability and Line 129 being versatile under various conditions. Also, Line 129 was superior in vegetative and reproductive vigor and protein content.

### GGE biplot for the studied traits

For a variety of soybean agronomic variables, the GGE biplot showed that genotype performances varied across different environments. According to the experimental findings, Line 105 and Line 129 showed different patterns of adaptability for the morphological traits: Line 105 seems to function better in a variety of settings (broad adaptation), while Line 129 tends to function better in particular circumstances (specific adaptation). Breeding programs demand this kind of isolation [49]. Giza 22 was characterized by seed size and yield across several environments, suggesting its efficient resource utilization and seed-filling capacity [50]. Seed yield per plant biplot indicated a very strong positive correlation in all environments, especially Environments 2 and 5, with Environments 3 and 6. The similarities indicate consistent yield-supporting conditions, like equal watering or plant density [51]. Giza 22’s yield in Environment 4, Line 127’s yield in Environment 5, and Line 129’s performance in Environments 3 and 6 pinpoint genotype-specific strategies to achieve maximum yield, whether in the form of high pod counts, seed weight, or loss in empty pods. For SY/ha, the proximity between Env3 and Env5, and Environments2 and Env6, suggests shared productivity-enhancing traits like good stand establishment or minimal biotic stress [49]. Also, the high performance of Line 127 in Environments 1 and 4 suggests field-level robustness and resilience. Line 129’s versatility at a high level in Environments 2, 3, 5, and 6 also contributes to its adaptability as a widely adopted, high-yielding genotype [52]. Line 129 also contains valuable genetic potential, particularly in protein content maximization under various conditions of the environment. Plant breeders to achieve stability of seed quality need to target these genotypes, especially Environments 2 and 5-like environments [53].

### The mean performance versus the stability of the studied characteristics

For important morphological features, the investigated genotypes showed significant variance, according to mean stability and performance analysis across six settings. Line 101’s genotype demonstrated better mean germination performance, indicating more initial vigour under the conditions under investigation. Line 105 demonstrated the best germination performance, suggesting that these lines may have growth limitations during the early establishing phases [54]. Giza 22 had the earliest flowering and maturity, hence being advantageous for deployment under double-cropping or short-season conditions. Line 129 was inclined to delayed flowering and maturity, which is desirable under situations of long growing seasons and thereby inclined to have long durations of biomass accumulation and yield [55]. Line 129 also recorded the greatest plant height, which is often attributed to enhanced competitive power and biomass production, whereas Line 127 was the shortest among the test lines. As for pubescence, Line 129 had the highest number of hairs, which can be related to high resistance to biotic and abiotic stresses [49]. Defoliation levels also distinguished genotypes, with Giza 22 being the most defoliated and Line 105 having the least defoliation, a trait that could be used as an indicator of resistance to pests or retention of leaves under stress [56]. Stability, expressed as deviation from the performance axis in biplot, showed that most genotypes exhibited high adaptability [51]. Line 127 was the least stable for germination, even at overall genotype stability. Conversely, Giza 22 consistently demonstrated superiority in stability of flowering and maturity characteristics, indicating it is well-buffered against environmental fluctuations during these stages [57]. Line 129 was also stable for these characters, which also supports it as a versatile line. Surprisingly, Line 127, being the shortest in plant height, was most stable for this trait, suggesting a phenotypic stability in any environmental condition [57]. Line 129 again performed well in performance and stability for plant height, confirming its agronomic potential. For environments, the fourth and sixth were most stable for plant height, reflecting good conditions for growth consistency.

Comparison of mean performance against stability for the yield-related characteristics highlighted also a significant genotype-by-environment interactions, as well as selection of superior soybean genotypes with superior agronomic characteristics. The highest mean performance for branches was Giza 111, indicating superior branching ability and possibly more assimilate allocation to reproductive locations [37]. Line 105 had minimum stability for the trait, suggesting that its performance was highly variable across the different environments [37, 58]. Seed size varied with genotypes, and Giza 22 was superior to the others in mean performance as well as being the most stable. Such stability across conditions is a favorable trait in Giza 22, where seed uniformity is an issue. Line 127 was the least stable with very high variation in expression of seed size across environments. In pod number, Lines 127 and 105 are traced closely along the performance vector, which reveals high average performance [37]. However, Giza 22 saw a decrease in pod production, which, in some circumstances, may limit its potential yield. The most stable genotypes, according to stability analysis, were Giza 111, Giza 22, and Line 127; Line 129, on the other hand, showed low stability, indicating significant environmental sensitivity [5]. The 100-seed weight trait also revealed Giza 22’s better performance, as revealed by its location on the positive performance axis. Lines 105 and 129 were lower in mean values. The highest stability was expressed by Giza 111, revealing constant expression of seed weight across all environments tested [40]. Per plant and hectare seed yield test results indicated Line 127 to be the best performer, followed by Giza 22, reflecting their high individual plant productivity potential. Per plant yield was lowest in Line 105. Line 129 and Giza 22 demonstrated yield consistency, reflecting its stability under changing growing conditions [59].

For protein content, the Line 129 genotype had superior mean performance, having been positioned farthest along the positive direction of the average environment coordination (AEC) abscissa, reflecting larger mean values [18, 60]. The lowest protein content was that of Giza 111, positioned at the opposite end of the axis. For stability, Line 129 and Giza 111 were the most stable genotypes, as indicated by their proximity to the AEC ordinate, with minimal interaction with environmental variation. Line 105, being furthest from the AEC ordinate, was the most unstable for protein content, with a high genotype-by-environment interaction that could limit its reliability across diverse environments [61]. For oil content, Line 105 showed the highest mean performance, whereas Giza 111 once more had the lowest figures. Contrary to protein content, the stability pattern differed; Line 105 and Giza 111 were the most stable genotypes for oil content, as they were nearly parallel to the AEC line. The most unstable was Line 129, implying poor consistency in oil accumulation across environments. The mean stability and performance analysis of protein and oil content characteristics revealed clear differences between the soybean genotypes according to their chemical composition and environmental suitability. Overall, these results point out the sophistication of genotype-by-environment interactions for soybean quality traits. Genotypes like Line 129 and Giza 111 offer contrasting profiles, high performance vs. high stability, which calls for selecting genotypes based on the target breeding objective, whether it is for performance, stability, or an intermediate degree of both [61].

## Conclusion

The evaluation of soybean genotypes across different environments revealed significant variation in performance and stability for a wide range of traits, including morphological characteristics, yield contributing characteristics, and chemical content (protein and oil percentage). Our findings revealed that among genotypes, Line 129 consistently demonstrated higher mean performance for a variety of traits, including protein content, plant height, number of hairs, and days to maturity, and also high stability for the majority of traits across environments. The MTSI analysis confirmed Line 129 as the most stable genotype in general and, therefore, a good option for breeding programs targeting multi-trait resilience against shifting environmental conditions. Therefore, according to our results, Giza line 129 is a superior option for adding to breeding programs for soybean production, quality, and stability. These results provide a sound foundation for future breeding programs to improve both yield and quality in different environments. Future research must focus on integrating physiological measurements, e.g., water-use efficiency, stomatal conductance, chlorophyll fluorescence, and root architecture, with genomic and transcriptomic analyses for identifying candidate genes and pathways involved in the response to drought. This integrative approach will increase the selection and breeding of soybean genotypes that have agronomic tolerance and demonstrated physiological tolerance to water-stress conditions, facilitating sustainable soybean production amidst climate change pressure.

## Supplementary Information

Supplementary Material 1.

## Data Availability

The data sets generated and analyzed in this study are available from the corresponding author on request.
