# Supplementary material for: Identification of high-yielding and stable Egyptian soybean genotypes for breeding across varied environments
Source: BMC Plant Biol. 2026 Jan 9;26:168. doi: 10.1186/s12870-025-07942-4 (PMC12849685; doi:10.1186/s12870-025-07942-4)
Supplement: Supplementary file 1 — Supplementary Material 1. [file 12870_2025_7942_MOESM1_ESM.docx]

**Table S1** Meteorology of the experimental area during the 2023 and 2024 seasons.

| Month | HC Air temperature [°C] | | | Solar radiation | HC Relative humidity [%] | | |
| --- | --- | --- | --- | --- | --- | --- | --- |
|  | min | Max | Avg | Dgt [W/m^2^] | Min | Max | Avg |
| 2023 | | | | | | | |
| May | 21.74 | 33.48 | 27.61 | 105.38 | 50.48 | 88.53 | 69.51 |
| June | 22.37 | 42.03 | 32.20 | 108.13 | 66.69 | 89.84 | 78.27 |
| July | 20.55 | 36.61 | 28.58 | 109.01 | 67.33 | 90.00 | 78.67 |
| Aug. | 19.76 | 32.40 | 26.08 | 105.35 | 75.23 | 88.91 | 82.07 |
| Spt. | 15.88 | 31.72 | 23.80 | 91.66 | 66.61 | 89.43 | 78.02 |
| Oct. | 14.71 | 28.08 | 21.39 | 102.40 | 35.65 | 93.64 | 64.65 |
| 2024 | | | | | | | |
| May | 25.32 | 32.02 | 28.67 | 107.98 | 46.93 | 86.81 | 66.87 |
| June | 26.00 | 43.74 | 34.87 | 113.65 | 75.17 | 85.79 | 80.48 |
| July | 19.07 | 35.88 | 27.47 | 104.28 | 65.37 | 85.17 | 75.27 |
| Aug. | 19.49 | 33.11 | 26.30 | 107.99 | 77.24 | 84.74 | 80.99 |
| Spt. | 18.51 | 30.33 | 24.42 | 95.25 | 71.65 | 87.25 | 79.45 |
| Oct. | 15.23 | 30.45 | 22.84 | 105.16 | 40.19 | 92.61 | 66.40 |

**Table S2** Physical and chemical analysis of the experimental soil before planting in the 2023 and 2024 seasons.

| Parameters | 2022/2023 Season | 2023/2024 Season |
| --- | --- | --- |
| **Mechanical Analysis** |  |  |
| Sand% | 7.09 | 7.07 |
| Silt% | 31.80 | 31.61 |
| Clay% | 61.11 | 61.32 |
| Soil Texture | Clay | Clay |
| **Chemical Analysis** |  |  |
| pH | 7.56 | 7.58 |
| Organic Matter (OM)% | 1.3 | 1.32 |
| Electrical Conductivity  (EC) mmhos/cm | 1.69 | 1.93 |
| Soil Porosity (Sp)% | 83.8 | 84.3 |
| **Soluble Anions** |  |  |
| Bicarbonate (HCO₃⁻) meq/L | 0.76 | 0.84 |

**Table S3** ANOVA summary for soybean traits across water stress levels, genotypes, and their interactions.

| **S.O.V.** | **DF** | **Germination** | **DH** | **DM** | **PH** | **NH** | **Defoliation** | **NB** | **SZ** | **NP/P** | **NEP** | **HSW** | **SY/P** | **SY/ha** | **Protein** | **Oil** |
| --- | --- | --- | --- | --- | --- | --- | --- | --- | --- | --- | --- | --- | --- | --- | --- | --- |
| Water Stress | 2 | 3.80 ns | 4.87 * | 195.49 ** | 2888.89 ** | 46.82 ** | 19.00 ** | 3.02 * | 490.83 ** | 31.32 ns | 251.57 ** | 18.24 ** | 69.77 ** | 521,837.55 ** | 260.85 ** | 23.40 ** |
| Genotype | 4 | 77.08 ** | 35.71 ** | 30.66 ** | 134.50 ** | 49.56 ** | 38.93 ** | 3.10 ** | 75.12 ** | 42.90 ** | 0.67 ns | 11.55 ** | 11.33 ** | 35,944.10 ** | 16.14 ** | 17.90 ** |
| Water stress × Genotype | 8 | 7.88 ns | 2.39 * | 7.79 ** | 112.50 ** | 0.93 ns | 50.65 ** | 0.35 * | 94.99 ** | 9.40 ns | 4.34 ** | 1.60 ns | 4.48 ** | 35,801.41 ** | 9.99 *** | 5.74 *** |
| Rep × Water stress (main error) | 4 | 2.47 | 1.03 | 0.66 | 3.89 | 1.96 | 0.37 | 0.33 | 15.37 | 5.09 | 0.26 | 0.57 | 0.33 | 1,486.64 | 0.94 | 0.11 |
| Residual (subplot error) | 24 | 3.66 | 1.02 | 1.39 | 15.69 | 2.72 | 4.23 | 0.17 | 19.08 | 14.68 | 0.35 | 0.97 | 0.36 | 1,676.63 | 1.25 | 0.31 |

**ns = non-significant (p > 0.05); * = significant (p ≤ 0.05); ** = highly significant (p ≤ 0.01).**

**Table S4** AMMI analysis of the interaction effects of genotype and environment on soybean morphological features.

| Traits | Source | Df | Sum Sq | Mean Sq | F value | Pr(>F) | Proportion | Accumulated |
| --- | --- | --- | --- | --- | --- | --- | --- | --- |
| Germination | ENV | 5 | 17.43 | 3.48 | 1.2 | 3.63E-01 | - | - |
|  | REP | 12 | 34.66 | 2.88 | 0.79 | 6.54E-01 | - | - |
|  | GEN | 4 | 2483.82 | 620.95 | 170.64 | 9.80E-28 | - | - |
|  | GEN*ENV | 20 | 125.511 | 6.27 | 1.72 | 6.23E-02 | - | - |
|  | PC1 | 8 | 81.20 | 10.15 | 2.79 | 1.28E-02 | 95.54 | 95.54 |
|  | PC2 | 6 | 24.23 | 4.03 | 1.11 | 3.70E-01 | 3.1 | 98.64 |
|  | PC3 | 4 | 11.98 | 2.99 | 0.82 | 5.18E-01 | 1.02 | 99.66 |
|  | PC4 | 2 | 8.08 | 4.04 | 1.11 | 3.37E-01 | 0.34 | 100.0 |
|  | Residuals | 60 | 174.66 | 3.63 | - | - | - | - |
|  | Total | 89 | 2961.6111 | 27.17 | - | - | - | - |
| DF | ENV | 5 | 34.76 | 6.95 | 3.91 | 2.45E-02 | - | - |
|  | REP | 12 | 21.33 | 1.77 | 1.29 | 2.53E-01 | - | - |
|  | GEN | 4 | 1099.55 | 274.88 | 199.91 | 2.79E-29 | - | - |
|  | GEN*ENV | 20 | 30.84 | 1.54 | 1.121 | 3.60E-01 | - | - |
|  | PC1 | 8 | 25.00 | 3.12 | 2.27 | 3.80E-02 | 98.91 | 98.91 |
|  | PC2 | 6 | 4.65 | 0.77 | 0.56 | 7.59E-01 | 0.96 | 99.87 |
|  | PC3 | 4 | 0.81 | 0.20 | 0.15 | 9.62E-01 | 0.11 | 99.98 |
|  | PC4 | 2 | 0.36 | 0.18 | 0.13 | 8.78E-01 | 0.02 | 100.0 |
|  | Residuals | 60 | 66 | 1.37 | - | - | - | - |
|  | Total | 89 | 1283.34 | 11.77 | - | - | - | - |
| DM | ENV | 5 | 700.72 | 140.14 | 323.41 | 2.32E-12 | - | - |
|  | REP | 12 | 5.2 | 0.43 | 0.35 | 9.73E-01 | - | - |
|  | GEN | 4 | 2364.73 | 591.18 | 482.59 | 4.22E-38 | - | - |
|  | GEN*ENV | 20 | 133.66 | 6.68 | 5.45 | 7.49E-07 | - | - |
|  | PC1 | 8 | 121.68 | 15.21 | 12.42 | 0.00E+00 | 97.45 | 97.45 |
|  | PC2 | 6 | 9.19 | 1.53 | 1.25 | 2.98E-01 | 2.16 | 99.61 |
|  | PC3 | 4 | 1.58 | 0.39 | 0.32 | 8.63E-01 | 0.27 | 99.88 |
|  | PC4 | 2 | 1.20 | 0.60 | 0.49 | 6.15E-01 | 0.12 | 100.0 |
|  | Residuals | 60 | 58.8 | 1.22 | - | - | - | - |
|  | Total | 89 | 3396.78 | 31.16 | - | - | - | - |
| PH | ENV | 5 | 12839.16 | 2567.83 | 200.96 | 3.90E-11 | - | - |
|  | REP | 12 | 153.33 | 12.77 | 0.82 | 6.27E-01 | - | - |
|  | GEN | 4 | 9604.44 | 2401.11 | 154.35 | 9.16E-27 | - | - |
|  | GEN*ENV | 20 | 1728.88 | 86.44 | 5.55 | 5.77E-07 | - | - |
|  | PC1 | 8 | 1305.01228 | 163.12 | 10.49 | 0.00E+00 | 90.54 | 90.54 |
|  | PC2 | 6 | 366.60037 | 61.10 | 3.93 | 2.80E-03 | 6.93 | 97.47 |
|  | PC3 | 4 | 57.18881 | 14.29 | 0.92 | 4.60E-01 | 2.01 | 99.48 |
|  | PC4 | 2 | 0.08743 | 0.043 | 0.00 | 1.00E+00 | 0.52 | 100.0 |
|  | Residuals | 60 | 746.66 | 15.55 | - | - | - | - |
|  | Total | 89 | 26801.38 | 245.88 | - | - | - | - |

DF = Days to flowering, DM = Days to maturity and PH = Plant height.

**Table S4** Continued.

| Traits | Source | Df | Sum Sq | Mean Sq | F value | Pr(>F) | Proportion% | Accumulative% |
| --- | --- | --- | --- | --- | --- | --- | --- | --- |
| NH | ENV | 5 | 199.7 | 39.94 | 7.048 | 2.72E-03 | - | - |
|  | REP | 12 | 68 | 5.66 | 1.64 | 1.10E-01 | - | - |
|  | GEN | 4 | 3647.28 | 911.82 | 264.72 | 4.68E-32 | - | - |
|  | GEN*ENV | 20 | 34.57 | 1.72 | 0.5 | 9.52E-01 | - | - |
|  | PC1 | 8 | 23.54 | 2.94 | 0.85 | 5.64E-01 | 99.54 | 99.54 |
|  | PC2 | 6 | 9.09 | 1.51 | 0.44 | 8.48E-01 | 0.38 | 99.92 |
|  | PC3 | 4 | 1.90 | 0.47 | 0.14 | 9.66E-01 | 0.06 | 99.98 |
|  | PC4 | 2 | 0.02 | 0.01 | 0.00 | 1.00E+00 | 0.02 | 100.0 |
|  | Residuals | 60 | 165.33 | 3.44 | - | - | - | - |
|  | Total | 89 | 4149.47 | 38.06 | - | - | - | - |
| Defoliation | ENV | 5 | 91.66 | 18.33 | 11.03 | 3.71E-04 | - | - |
|  | REP | 12 | 19.93 | 1.66 | 0.4 | 9.51E-01 | - | - |
|  | GEN | 4 | 4482.22 | 1120.55 | 270.65 | 1.65E-22 | - | - |
|  | GEN*ENV | 20 | 170.39 | 10.02 | 2.42 | 1.74E-02 | - | - |
|  | PC1 | 8 | 177.33 | 22.16 | 5.35 | 4.00E-04 | 98.58 | 98.58 |
|  | PC2 | 6 | 20.44 | 3.40 | 0.82 | 5.63E-01 | 1.27 | 99.85 |
|  | PC3 | 4 | 8.50 | 2.12 | 0.51 | 7.28E-01 | 0.10 | 99.95 |
|  | PC4 | 2 | 0.61 | 0.30 | 0.070 | 9.32E-01 | 0.05 | 100.0 |
|  | Residuals | 60 | 120.06 | 4.14 | - | - | - | - |
|  | Total | 89 | 5091.19 | 58.51 | - | - | - | - |

NH = Number of hairs.

**Table S5** Yield-related parameter variations in genotypes, environments, and their interactions were analyzed via AMMI analysis

| Traits | Source | Df | Sum Sq | Mean Sq | F value | Pr(>F) |  |  |
| --- | --- | --- | --- | --- | --- | --- | --- | --- |
| NB | ENV | 5 | 9.62 | 1.92 | 11 | 3.76E-04 | - | - |
|  | REP | 12 | 2.1 | 0.17 | 1.61 | 0.12 | - | - |
|  | GEN | 4 | 27.92 | 6.98 | 64.38 | 2.30E-17 | - | - |
|  | GEN*ENV | 20 | 4.56 | 0.22 | 2.1 | 0.02 | - | - |
|  | PC1 | 8 | 3.04 | 0.38 | 3.52 | 0.003 | 96 | 96 |
|  | PC2 | 6 | 1.51 | 0.25 | 2.33 | 0.04 | 2.47 | 98.47 |
|  | PC3 | 4 | 0.551020 | 0.13 | 1.27 | 0.29 | 1.03 | 99.5 |
|  | PC4 | 2 | 0.019040 | 0.009 | 0.09 | 0.9141 | 0.5 | 100.0 |
|  | Residuals | 60 | 4.55 | 0.1 | - | - | - | - |
|  | Total | 89 | 53.9 | 0.52 | - | - | - | - |
| SZ | ENV | 5 | 1273.46 | 254.69 | 7.79 | 1.78E-03 | - | - |
|  | REP | 12 | 392.07 | 32.67 | 1.3 | 0.2456 | - | - |
|  | GEN | 4 | 5814.06 | 1453.51 | 58.16 | 8.20E-18 | - | - |
|  | GEN*ENV | 20 | 1548.56 | 77.42 | 3.09 | 6.88E-04 | - | - |
|  | PC1 | 8 | 1068.30 | 133.53 | 5.34 | 1.00e-04 | 86.32 | 86.32 |
|  | PC2 | 6 | 391.91 | 65.31 | 2.61 | 2.86e-02 | 12.35 | 98.67 |
|  | PC3 | 4 | 74.27 | 18.56 | 0.74 | 0.5694 | 0.9 | 99.57 |
|  | PC4 | 2 | 14.06 | 7.03 | 0.28 | 0.7570 | 0.43 | 100.0 |
|  | Residuals | 60 | 1199.52 | 24.99 | - | - | - | - |
|  | Total | 89 | 11776.26 | 108.03 | - | - | - | - |

NB = Number of branches and SZ = Seed size.

**Table S5** Continued

| Traits | Source | DF | Sum Sq | Mean Sq | F value | Pr(>F) | Proportion% | Accumulative% |
| --- | --- | --- | --- | --- | --- | --- | --- | --- |
|  | ENV | 5 | 108.36 | 21.67 | 1.1 | 0.4 | - | - |
| NP/P | REP | 12 | 234.95 | 19.579 | 0.95 | 0.5 | - | - |
|  | GEN | 4 | 4795.17 | 1198.79 | 58.49 | 7.32E-18 | - | - |
|  | GEN*ENV | 20 | 405.21 | 20.26 | 0.98 | 0.49 | - | - |
|  | PC1 | 8 | 271.15 | 33.89 | 1.65 | 0.13 | 94.99 | 94.99 |
|  | PC2 | 6 | 110.97 | 18.49 | 0.9 | 0.50 | 4.23 | 99.22 |
|  | PC3 | 4 | 15.46759 | 3.86 | 0.19 | 0.94 | 0.66 | 99.88 |
|  | PC4 | 2 | 7.61873 | 3.80 | 0.19 | 0.82 | 0.12 | 100.0 |
|  | Residuals | 60 | 983.67 | 20.49 | - | - | - | - |
|  | Total | 89 | 6932.58 | 63.6 | - | - | - | - |
| NEP | ENV | 5 | 924.7 | 184.94 | 240.91 | 1.33E-11 | - | - |
|  | REP | 12 | 9.21 | 0.76 | 1.709 | 0.09 | - | - |
|  | GEN | 4 | 7.81 | 1.95 | 4.351 | 0.004 | - | - |
|  | GEN*ENV | 20 | 59.09 | 2.95 | 6.579 | 4.76E-08 | - | - |
|  | PC1 | 8 | 55.64 | 6.95 | 15.49 | < 0.0001 | 84.59 | 84.59 |
|  | PC2 | 6 | 2.84 | 0.47 | 1.05 | 0.40 | 14.36 | 98.95 |
|  | PC3 | 4 | 0.48 | 0.12 | 0.27 | 0.89 | 0.1 | 99.05 |
|  | PC4 | 2 | 0.12 | 0.06 | 0.14 | 0.86 | 0.95 | 100.0 |
|  | Residuals | 60 | 21.55 | 0.44 | - | - | - | - |
|  | Total | 89 | 1081.47 | 9.92 | - | - | - | - |
| HSW | ENV | 5 | 53.4 | 10.68 | 8.001 | 0.001 | - | - |
|  | REP | 2 | 16.01 | 1.33 | 1.13 | 0.35 | - | - |
|  | GEN | 4 | 358 | 89.5 | 76.19 | 3.52E-20 | - | - |
|  | GEN*ENV | 20 | 27.6 | 1.38 | 1.175 | 0.31 | - | - |
|  | PC1 | 8 | 14.77 | 1.84 | 1.57 | 0.15 | 95.93 | 95.93 |
|  | PC2 | 6 | 9.89 | 1.64 | 1.40 | 0.23 | 3.26 | 99.19 |
|  | PC3 | 4 | 2.67 | 0.66 | 0.570 | 0.68 | 0.78 | 99.97 |
|  | PC4 | 2 | 0.25 | 0.12 | 0.11 | 0.89 | 0.0.03 | 100.0 |
|  | Residuals | 60 | 56.37 | 1.17 | - | - | - | - |
|  | Total | 109 | 539.01 | 4.94 | - | - | - | - |
| SY/P | ENV | 5 | 278.17 | 55.63 | 106.87 | 1.60E-09 | - | - |
|  | REP | 12 | 6.24 | 0.52 | 1.6 | 0.12 | - | - |
|  | GEN | 4 | 66.46 | 16.61 | 51.25 | 9.67E-17 | - | - |
|  | GEN*ENV | 20 | 73.79 | 3.68 | 11.38 | 4.52E-12 | - | - |
|  | PC1 | 8 | 69.24 | 8.65 | 26.70 | 0.00 | 88.08 | 88.08 |
|  | PC2 | 6 | 3.62 | 0.60 | 1.87 | 0.10 | 10.98 | 99.06 |
|  | PC3 | 4 | 0.85 | 0.21 | 0.66 | 0.62 | 0.74 | 99.8 |
|  | PC4 | 2 | 0.06 | 0.03 | 0.10 | 0.90 | 0.2 | 100.0 |
|  | Residuals | 60 | 15.56 | 0.32 | - | - | - | - |
|  | Total | 89 | 514.04 | 4.71 | - | - | - | - |

NP/P = Number of pods per plant, NEP = Number of empty pods, HSW = Hundred seed weight and SY/P = Seed yield per plant.

**Table S5** Continued

| Traits | Source | Df | Sum Sq | Mean Sq | F value | Pr(>F) | Proportion% | | Accumulated% | |
| --- | --- | --- | --- | --- | --- | --- | --- | --- | --- | --- |
| SY/ha | ENV | 5 | 1,985,93 | 397,186.41 | 86.91 | 5.33E-09 | - | - | |  |
|  | REP | 12 | 54,835.72 | 4,569.64 | 3.17 | 2.17E-03 | - | - | |  |
|  | GEN | 4 | 229,707.20 | 57,426.81 | 39.844 | 1.09E-14 | - | - | |  |
|  | GEN*ENV | 20 | 537,251.50 | 26,862.58 | 18.638 | 3.21E-16 | - | - | |  |
|  | PC1 | 8 | 511,169.7 | 63,896.21 | 44.33 | 0.00 | 66.65 | 66.65 | |  |
|  | PC2 | 6 | 24,691.6 | 4,115.26 | 2.86 | 0.01 | 30.2 | 96.85 | |  |
|  | PC3 | 4 | 1,383.08 | 345.77 | 0.24 | 0.91 | 3.12 | 99.97 | |  |
|  | PC4 | 2 | 7.23 | 3.61 | 0.00 | 1.00 | 0.03 | 100.0 | |  |
|  | Residuals | 60 | 69,182.50 | 1,441.30 | - | - | - | - | |  |
|  | Total | 89 | 3,414,161 | 31,322.57 | - | - | - | - | |  |

SY/F = Seed yield per hectare.

**Table S6** AMMI analysis of variance for protein and oil contents in different genotypes, environments and their interactions

| Traits |  | Source | Df | Sum Sq | Mean Sq | F value | Pr(>F) | Proportion% | Accumulative% |
| --- | --- | --- | --- | --- | --- | --- | --- | --- | --- |
| Protein |  | ENV | 5 | 988.77 | 197.75 | 57.82 | 5.57E-08 | - | - |
|  |  | REP | 12 | 41.03 | 3.41 | 3.09 | 2.61E-03 | - | - |
|  |  | GEN | 4 | 435.52 | 108.88 | 98.59 | 1.58E-22 | - | - |
|  |  | GEN*ENV | 20 | 163.94 | 8.19 | 7.42 | 7.16E-09 | - | - |
|  |  | PC1 | 8 | 126.28 | 15.78 | 14.29 | 0.00E+00 | 77.29 | 77.29 |
|  |  | PC2 | 6 | 34.94 | 5.82 | 5.27 | 3.00E-04 | 20.24 | 97.53 |
|  |  | PC3 | 4 | 2.61 | 0.65 | 0.59 | 6.71E-01 | 1.67 | 99.2 |
|  |  | PC4 | 2 | 0.09 | 0.04 | 0.04 | 9.60E-01 | 0.8 | 100 |
|  |  | Residuals | 69 | 53 | 1.1 | - | - | - | - |
|  |  | Total | 89 | 1846.24 | 16.93 | - | - | - | - |
| Oil |  | ENV | 5 | 96.96 | 19.39 | 27.9 | 3.25E-06 | - | - |
|  |  | REP | 2 | 8.33 | 0.69 | 2.24 | 2.40E-02 | - | - |
|  |  | GEN | 4 | 547.01 | 136.75 | 441.55 | 3.37E-37 | - | - |
|  |  | GEN*ENV | 20 | 90 | 4.5 | 14.53 | 4.48E-14 | - | - |
|  |  | PC1 | 8 | 70.73 | 8.84 | 28.55 | 0.00E+00 | 89.5 | 89.5 |
|  |  | PC2 | 6 | 18.9 | 3.15 | 10.18 | 0.00E+00 | 10.39 | 99.89 |
|  |  | PC3 | 4 | 0.29 | 0.07 | 0.24 | 9.14E-01 | 0.07 | 99.96 |
|  |  | PC4 | 2 | 0.06 | 0.03 | 0.1 | 9.05E-01 | 0.04 | 100 |
|  |  | Residuals | 48 | 14.86 | 0.3 | - | - | - | - |
|  |  | Total | 109 | 847.19 | 7.77 | - | - | - | - |

**Table S7** Estimated marginal means (EMMs) of the germination percentage, days to flowering, days to maturity, plant height, number of hairs and defoliation percentage of the five soybean genotypes in the six environments.

| Traits | Genotypes | Env1 | Env2 | Env3 | Env4 | Env5 | Env6 | SE | Lower.CL (Range) | Upper.CL (Range) |
| --- | --- | --- | --- | --- | --- | --- | --- | --- | --- | --- |
| Germination | Giza 111 | 83.3 | 82.3 | 83.3 | 79.3 | 82 | 81 | 3.94 | 71.6 - 75.6 | 87.1 - 91.1 |
|  | Giza 22 | 82.3 | 81 | 83.3 | 81 | 80.7 | 82.3 | 3.94 | 72.9 - 75.6 | 88.4 - 91.1 |
|  | Line 105 | 95.7 | 95.7 | 94.3 | 94 | 93.3 | 94.3 | 3.94 | 85.6 - 87.9 | 101.1 - 103.4 |
|  | Line 127 | 86.7 | 82.7 | 81.3 | 86 | 84.7 | 83.7 | 3.94 | 73.6 - 78.3 | 89.1 - 94.4 |
|  | Line 129 | 90 | 91.3 | 92.7 | 90.7 | 92.7 | 91.3 | 3.94 | 82.3 - 83.6 | 97.7 - 100.4 |
| DF | Giza 111 | 42 | 40.7 | 39 | 42 | 39.3 | 40.3 | 2.68 | 33.7 - 36.7 | 44.3 - 47.3 |
|  | Giza 22 | 40.3 | 39.3 | 38.7 | 41 | 38.7 | 38.3 | 2.68 | 33.1 - 35.1 | 43.6 - 46.3 |
|  | Line 105 | 45.3 | 46 | 46 | 45.7 | 45 | 44.7 | 2.68 | 39.4 - 40.1 | 49.9 - 51.3 |
|  | Line 127 | 44.3 | 42.7 | 42 | 44 | 41.7 | 43.3 | 2.68 | 36.4 - 38.1 | 46.9 - 49.6 |
|  | Line 129 | 48.7 | 49.7 | 49.3 | 49.3 | 48.7 | 49 | 2.68 | 43.4 - 44.1 | 53.9 - 54.9 |
| DM | Giza 111 | 121 | 118 | 116 | 122 | 119 | 117 | 1.72 | 113 - 119 | 120 - 125 |
|  | Giza 22 | 119 | 116 | 114 | 119 | 117 | 114 | 1.72 | 110 - 116 | 117 - 123 |
|  | Line 105 | 131 | 126 | 120 | 132 | 124 | 122 | 1.72 | 117 - 128 | 124 - 135 |
|  | Line 127 | 126 | 122 | 121 | 125 | 123 | 123 | 1.72 | 118 - 120 | 126 - 129 |
|  | Line 129 | 136 | 131 | 125 | 136 | 131 | 127 | 1.72 | 122 - 128 | 129 - 140 |
| PH | Giza 111 | 108.3 | 103.3 | 71.7 | 108.3 | 101.7 | 78.3 | 4.29 | 63.2 - 99.9 | 86.8 - 116.8 |
|  | Giza 22 | 101.7 | 95 | 65 | 103.3 | 98.3 | 68.3 | 4.29 | 56.6 - 89.9 | 76.8 - 111.8 |
|  | Line 105 | 116.7 | 105 | 91.7 | 118.3 | 96.7 | 83.3 | 4.29 | 74.9 - 109.9 | 100.1 - 126.8 |
|  | Line 127 | 95 | 86.7 | 71.7 | 98.3 | 85 | 65 | 4.29 | 56.6 - 78.2 | 73.4 - 106.8 |
|  | Line 129 | 118.3 | 116.7 | 106.7 | 121.7 | 111.7 | 103.3 | 4.29 | 94.9 - 98.2 | 111.8 - 130.1 |
| NH | Giza 111 | 14.67 | 17.33 | 17.67 | 14.67 | 16.33 | 16.67 | 5.67 | 3.54 - 6.21 | 25.8 - 28.8 |
|  | Giza 22 | 9.33 | 11.33 | 11.67 | 8.33 | 11.33 | 11 | 5.67 | -1.99 | 20.5 - 22.8 |
|  | Line 105 | 20.33 | 23.67 | 24.67 | 19.33 | 21.67 | 23 | 5.67 | 9.21 - 12.54 | 31.5 - 35.8 |
|  | Line 127 | 6.67 | 8.33 | 9.33 | 6.67 | 7.67 | 10 | 5.67 | -1.67 | 17.8 - 21.1 |
|  | Line 129 | 22.67 | 25.67 | 27.33 | 21.33 | 23.33 | 27 | 5.67 | 11.54 - 14.54 | 33.8 - 38.5 |
| Defoliation | Giza 111 | 17.33 | 19.67 | 22.67 | 18 | 20.33 | 21.33 | 7.11 | 3.41 - 5.74 | 31.3 - 35.3 |
|  | Giza 22 | 24.67 | 29.33 | 32.33 | 23.67 | 28.33 | 30 | 7.11 | 10.74 - 15.41 | 38.6 - 43.9 |
|  | Line 105 | 0 | 1.67 | 1.67 | 0 | 2.33 | 4 | 7.11 | -1.67 | 13.9 - 17.9 |
|  | Line 127 | 19.33 | 17.33 | 18.33 | 19.67 | 18 | 16.33 | 7.11 | 3.41 - 5.41 | 33.3 - 30.3 |
|  | Line 129 | 1.67 | 1.67 | 3.33 | 0 | 1 | 3 | 7.11 | -1.67 | 15.6 - 16.9 |

DF = Days to flowering, DM = Days to maturity, PH = Plant height and NH = Number of hairs.

**Table S8** Estimated marginal means (EMMs) for yield-related characteristics of the five soybean genotypes in six different environments.

| Traits | Genotypes | Env1 | Env2 | Env3 | Env4 | Env5 | Env6 | SE | Lower.CL (Range) | Upper.CL (Range) |
| --- | --- | --- | --- | --- | --- | --- | --- | --- | --- | --- |
| NB | Giza 111 | 2.66 | 2.53 | 3 | 2.53 | 2.53 | 2.86 | 0.5 | 1.53 - 1.66 | 3.53 - 4.00 |
|  | Giza 22 | 2.2 | 2.06 | 2.26 | 2.33 | 2 | 2.4 | 0.5 | 1.06 - 1.20 | 3.00 - 3.40 |
|  | Line 105 | 0.86 | 1.2 | 2.4 | 1.06 | 1.33 | 1.8 | 0.5 | -0.33 | 2.33 - 3.40 |
|  | Line 127 | 0.33 | 0.06 | 1.66 | 0.2 | 0.2 | 1.46 | 0.5 | 0.13 | 1.20 - 2.46 |
|  | Line 129 | 1.46 | 1 | 2.33 | 1.66 | 1.2 | 2.06 | 0.5 | 0.46 - 0.003 | 2.00 - 3.33 |
| SZ | Giza 111 | 42.2 | 37.7 | 28.7 | 37.4 | 41.1 | 38.7 | 7.89 | 13.20 - 26.71 | 44.2 - 57.7 |
|  | Giza 22 | 56.8 | 49.6 | 36 | 54.7 | 50.9 | 45.3 | 7.89 | 20.50 - 41.33 | 51.5 - 72.3 |
|  | Line 105 | 26.8 | 26.1 | 26.1 | 29.7 | 30.3 | 28.7 | 7.89 | 10.59 - 14.86 | 41.6 - 45.8 |
|  | Line 127 | 44.8 | 50.3 | 28.3 | 38.3 | 37.1 | 22.8 | 7.89 | 7.35 - 34.81 | 38.3 - 65.8 |
|  | Line 129 | 27.2 | 27.2 | 26.1 | 29 | 25 | 24.5 | 7.89 | 8.98 - 13.48 | 39.9 - 44.4 |
| NP/P | Giza 111 | 30.1 | 31.4 | 34.5 | 30.4 | 29.2 | 31.5 | 8.22 | 13.05 - 18.38 | 45.3 - 50.6 |
|  | Giza 22 | 29.4 | 26.2 | 31.9 | 28.9 | 26 | 25.6 | 8.22 | 9.50 - 15.81 | 41.7 - 48.0 |
|  | Line 105 | 45.6 | 48.4 | 48.3 | 42.3 | 41.8 | 43.6 | 8.22 | 25.69 - 32.31 | 57.9 - 64.5 |
|  | Line 127 | 31.3 | 30.6 | 32.6 | 33.3 | 34.2 | 30.8 | 8.22 | 14.53 - 18.11 | 46.8 - 50.3 |
|  | Line 129 | 41.7 | 46.4 | 45 | 40.4 | 50.8 | 46.5 | 8.22 | 24.32 - 34.68 | 56.6 - 66.9 |
| NEP | Giza 111 | 2.97 | 5.35 | 12.54 | 2.99 | 4.96 | 11.66 | 0.413 | 2.14 - 3.80 | 2.17 - 3.82 |
|  | Giza 22 | 3.06 | 4.92 | 11.96 | 2.97 | 4.87 | 10.41 | 0.413 | 2.23 - 3.89 | 2.14 - 3.79 |
|  | Line 105 | 3.28 | 6.49 | 9.4 | 3.24 | 6.11 | 8.97 | 0.413 | 2.45 - 4.10 | 2.41 - 4.07 |
|  | Line 127 | 3.26 | 5.53 | 12.48 | 3.35 | 5.84 | 11.8 | 0.413 | 2.44 - 4.09 | 2.52 - 4.17 |
|  | Line 129 | 3.43 | 7.13 | 9.84 | 3.26 | 7.31 | 9.9 | 0.413 | 2.61 - 4.26 | 2.43 - 4.08 |
| HSW | Giza 111 | 16.5 | 15 | 13.3 | 16.4 | 15.6 | 14.1 | 2.1 | 10.03-12.43 | 18.3-20.7 |
|  | Giza 22 | 17.2 | 17.2 | 14.1 | 17.2 | 17.2 | 16.1 | 2.1 | 11.99-13.06 | 20.2-21.3 |
|  | Line 105 | 11.5 | 10.8 | 10.6 | 12.3 | 12.2 | 11.5 | 2.1 | 7.34 - 7.35 | 15.6 - 15.6 |
|  | Line 127 | 16.7 | 15.7 | 14.2 | 15.9 | 14.6 | 14.4 | 2.1 | 10.24-12.57 | 18.5-20.8 |
|  | Line 129 | 12.8 | 11.8 | 11.7 | 12.9 | 10.9 | 11.5 | 2.1 | 7.41-8.70 | 15.7-16.9 |
| SY/P | Giza 111 | 16.4 | 13.4 | 11.1 | 16.4 | 12.8 | 10.9 | 0.431 | 10.0-15.5 | 11.7-17.2 |
|  | Giza 22 | 17.5 | 14.1 | 11.1 | 17 | 14 | 11.1 | 0.431 | 10.3-16.6 | 12.0-18.3 |
|  | Line 105 | 12.5 | 11.6 | 11 | 13.3 | 12.3 | 11.4 | 0.431 | 10.2-11.6 | 12.3-13.4 |
|  | Line 127 | 18.2 | 14.4 | 11.7 | 17.7 | 14 | 11.3 | 0.431 | 10.4-17.3 | 12.1-19.0 |
|  | Line 129 | 14.7 | 13.9 | 12.7 | 14 | 13.2 | 12.4 | 0.431 | 11.5-13.8 | 13.2-15.5 |
| SY/ha | Giza 111 | 567 | 459 | 384 | 542 | 438 | 366 | 29.5 | 341-543 | 390-592 |
|  | Giza 22 | 600 | 476 | 386 | 574 | 469 | 380 | 29.5 | 356-575 | 405-624 |
|  | Line 105 | 498 | 462 | 433 | 519 | 478 | 448 | 29.5 | 423-473 | 472-529 |
|  | Line 127 | 655 | 496 | 396 | 634 | 492 | 393 | 29.5 | 368-630 | 418-679 |
|  | Line 129 | 550 | 530 | 491 | 528 | 508 | 471 | 29.5 | 264-525 | 495-574 |

NB = Number of branches, SZ = Seed size, NP/P = Number of pods per plant, NEP = Number of empty pods, HSW = Hundred seed weight, SY/P = Seed yield per plant and SY/F = Seed yield per feddan.

**Table S9** Marginal means of the protein and oil levels of the five soybean genotypes in six distinct environments.

| Traits | Genotypes | Env1 | Env2 | Env3 | Env4 | Env5 | Env6 | SE | Lower CL (Range) | Upper CL (Range) |
| --- | --- | --- | --- | --- | --- | --- | --- | --- | --- | --- |
| Protein | Giza 111 | 35.5 | 32 | 26.8 | 33.9 | 30.6 | 25.6 | 1.14 | 33.2 - 37.7 | 24.5 - 29.1 |
|  | Giza 22 | 37.5 | 33.3 | 27 | 35.9 | 32.8 | 26.7 | 1.14 | 35.3 - 39.8 | 24.7 - 29.2 |
|  | Line 105 | 32.7 | 32.1 | 30.1 | 34.1 | 33.2 | 31.1 | 1.14 | 30.5 - 35.0 | 27.8 - 32.3 |
|  | Line 127 | 38.4 | 34.6 | 27.7 | 37.2 | 34.3 | 27.5 | 1.14 | 36.2 - 40.7 | 25.4 - 29.9 |
|  | Line 129 | 43.1 | 36.8 | 34.1 | 41.4 | 35.2 | 32.7 | 1.14 | 40.9 - 45.4 | 31.8 - 36.3 |
| Oil | Giza 111 | 14.8 | 14.4 | 16.4 | 14.2 | 13.8 | 15.7 | 1.25 | 12.4 - 17.3 | 14.0 - 18.9 |
|  | Giza 22 | 15.7 | 15 | 16.6 | 15 | 14.8 | 16.4 | 1.25 | 13.2 - 18.1 | 14.1 - 19.0 |
|  | Line 105 | 19.7 | 21.9 | 22.4 | 20.6 | 22.7 | 23.2 | 1.25 | 17.3 - 22.2 | 20.0 - 24.9 |
|  | Line 127 | 16.4 | 15 | 16.4 | 15.9 | 14.9 | 16.3 | 1.25 | 14.0 - 18.9 | 13.5 - 18.4 |
|  | Line 129 | 14.4 | 16.6 | 20.9 | 13.9 | 15.9 | 20.1 | 1.25 | 12.0 - 16.9 | 18.5 - 23.4 |


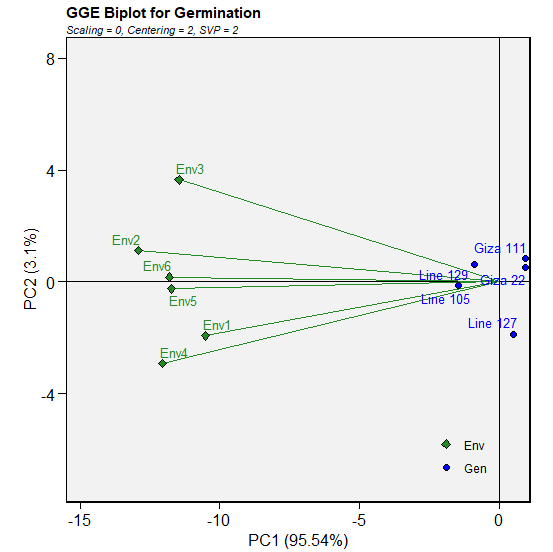

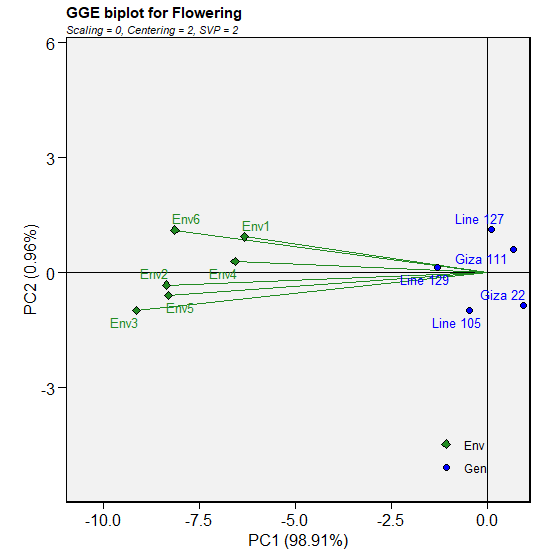

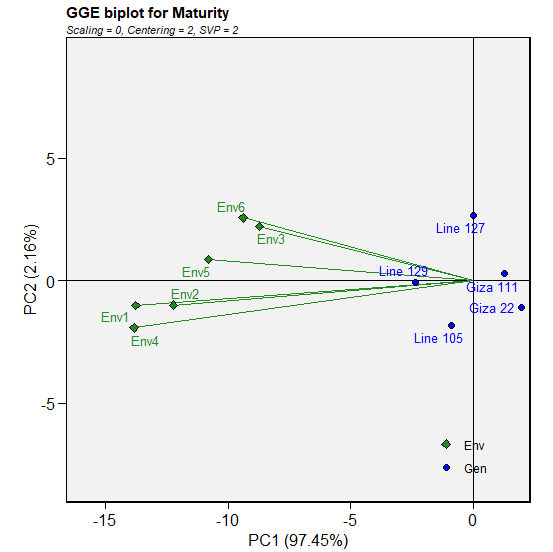

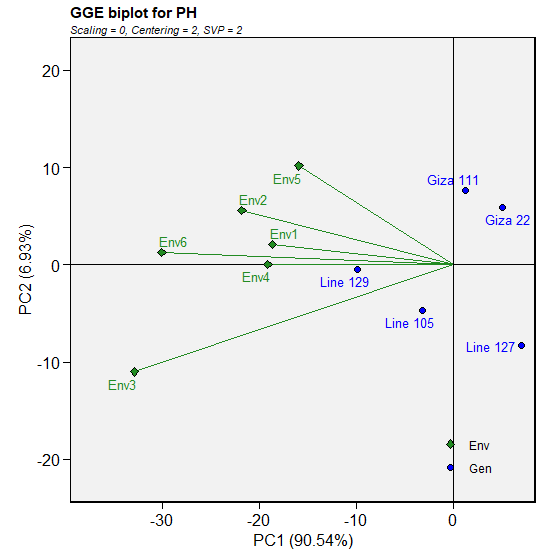


**Fig. S1** GGE biplot showing relationships among environments (green diamonds) and genotypes (blue circles) for the morphological characteristics (germination%, days to flowering and maturity, plant height (cm), number of hairs and defoliation%). Type of biplot: relationship biplot (environment vectors and genotype positions).


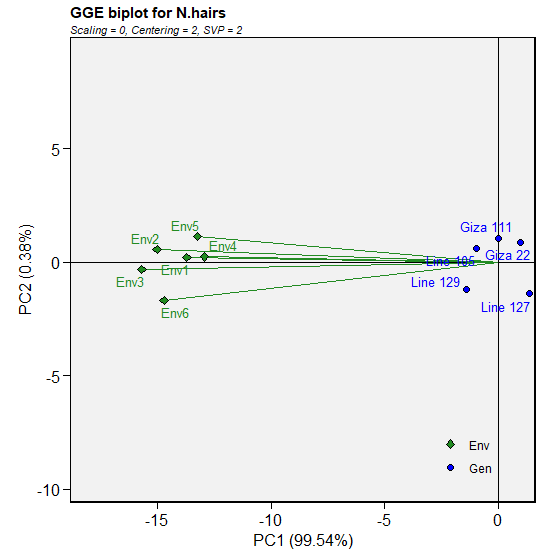

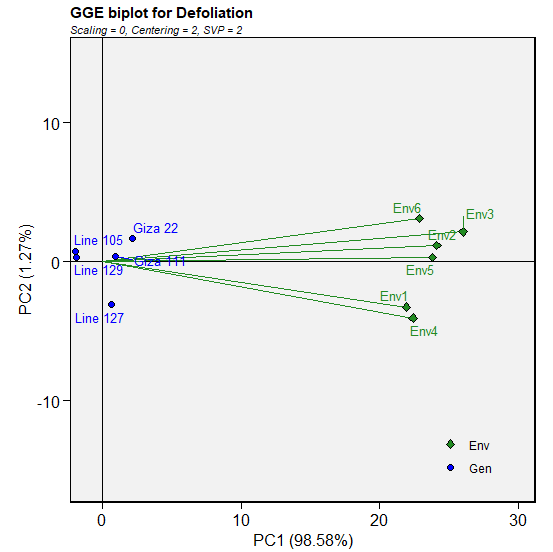


**Fig. S1** Continued.


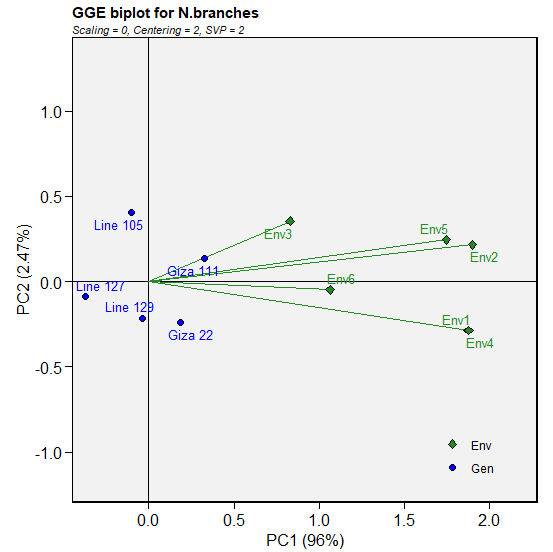

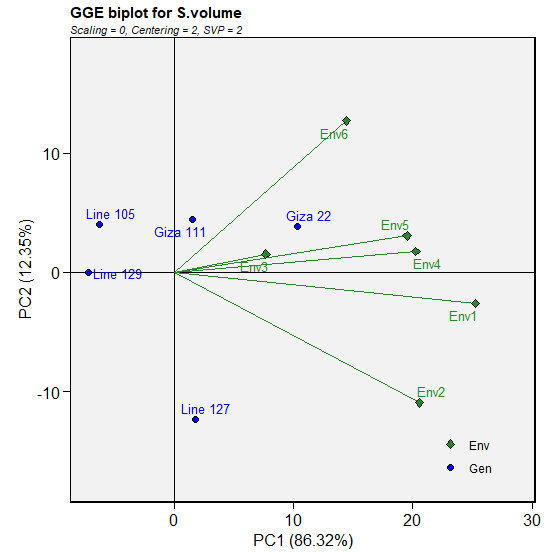

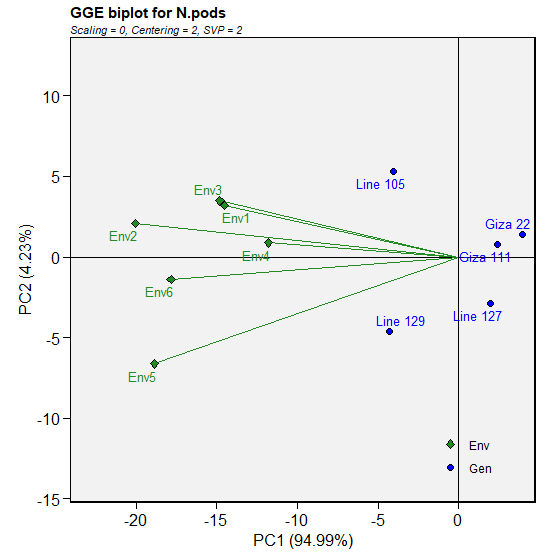

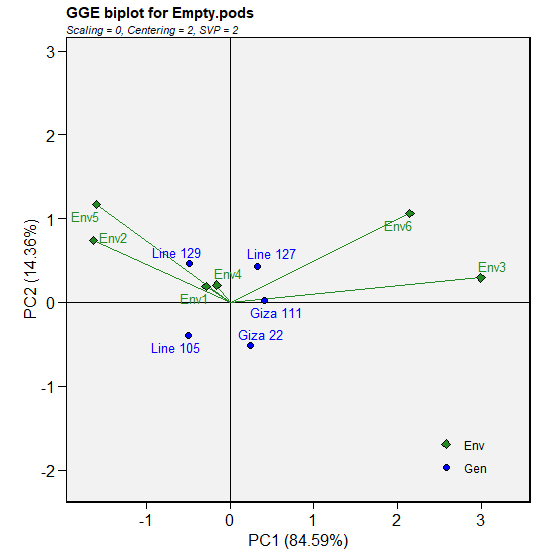


**Fig. S2** Relationships between genotypes (blue circles) and environments (green diamonds) for the yield-related parameters (number of branches, seed size (mm3), number of pods/plant, and number of empty pods) are displayed in a GGE biplot. Type of biplot: relationship biplot (environment vectors and genotype positions).


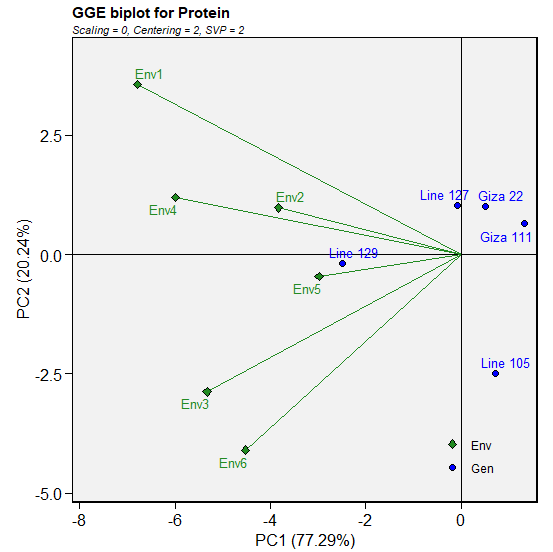

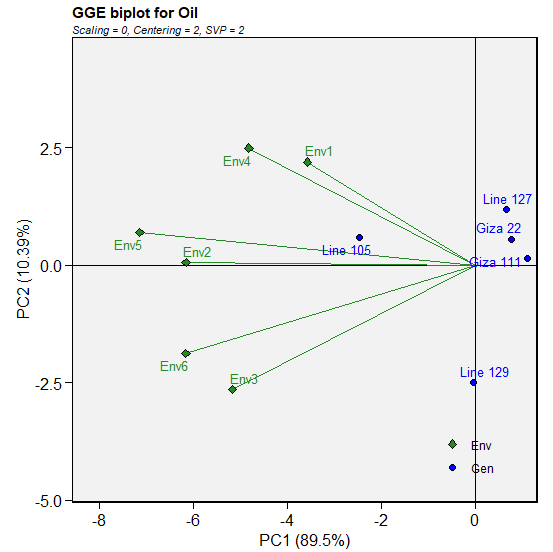


**Fig. S3** Protein and oil content GGE biplot. With the genotypes (blue dots) and environments (green triangles) situated on the first principal component (PC1) and second principal component (PC2), the biplot depicts the genotype‒environment interaction. The type of biplot: relationship biplot (environment vectors and genotype positions).


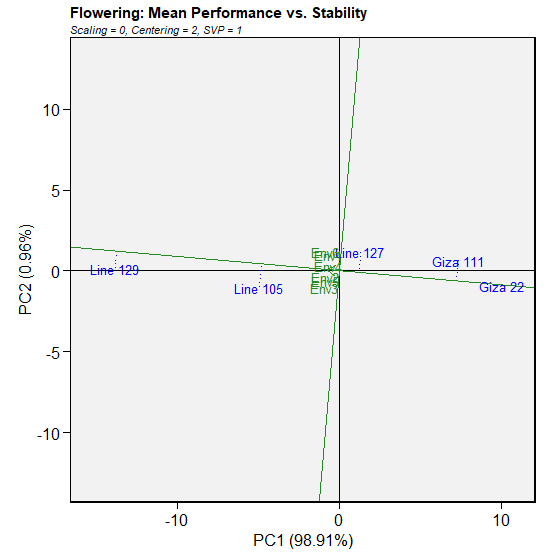

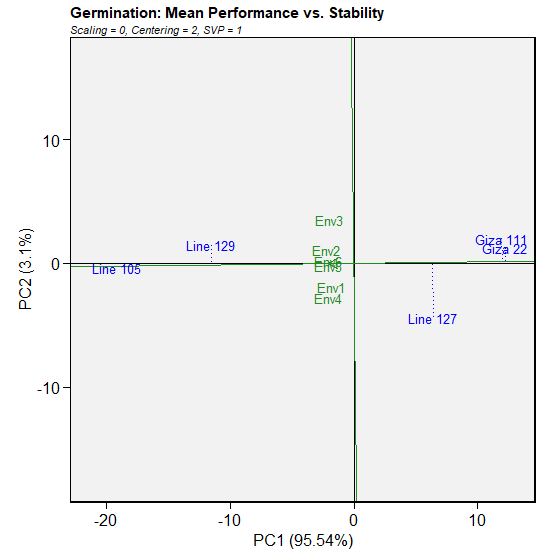


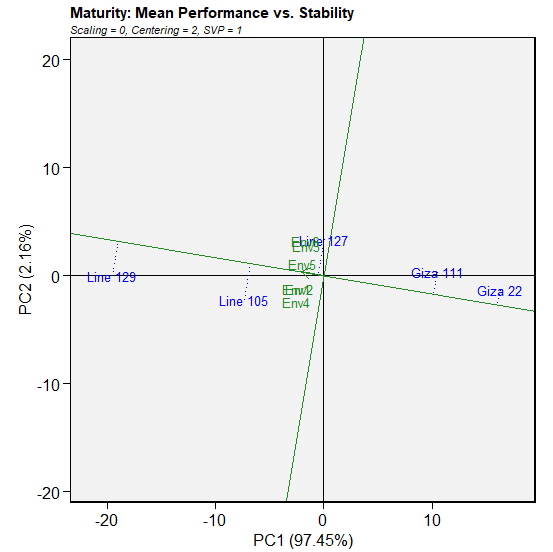

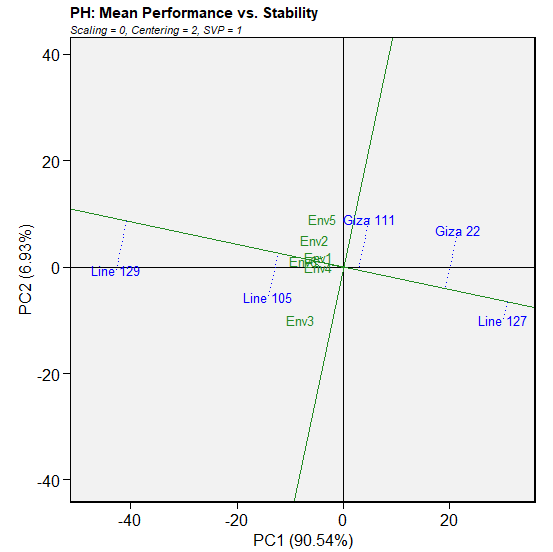


**Fig. S4** Mean performance vs. stability biplot for the morphological characteristics of five soybean genotypes and six environments. Biplot of the first two principal components (PC1 and PC2), where PC1 represents the mean performance and PC2 represents the stability. Average environment coordination (AEC) is used to derive genotype performance and stability. PH refers to plant height characteristic.


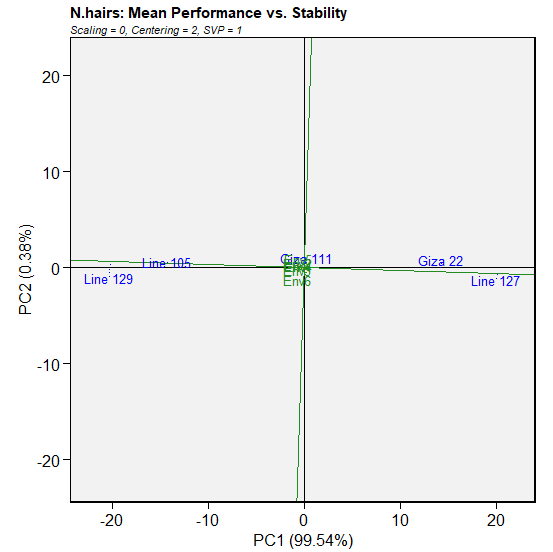

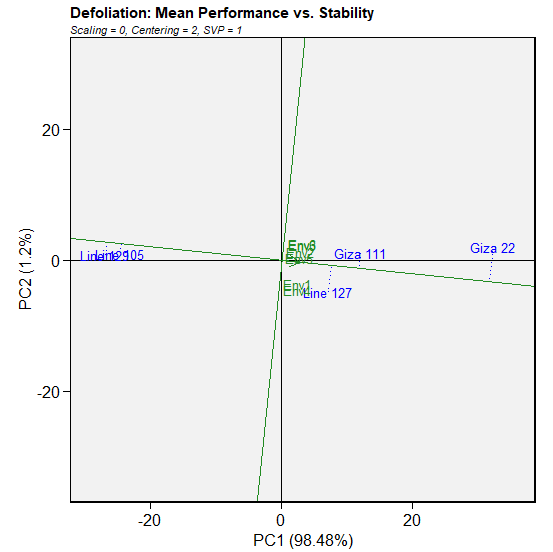


**Fig. S4** Continued


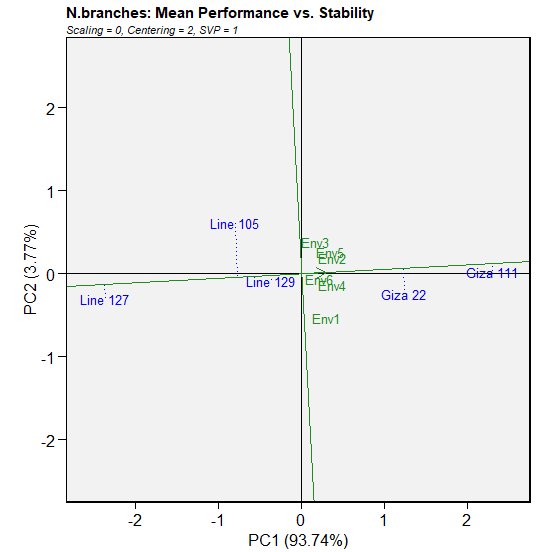

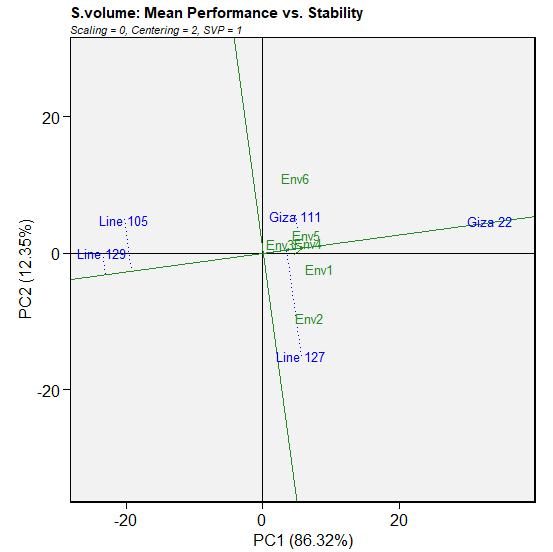


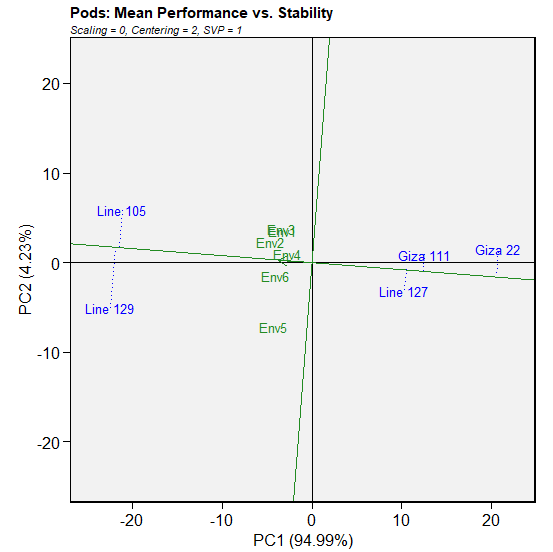

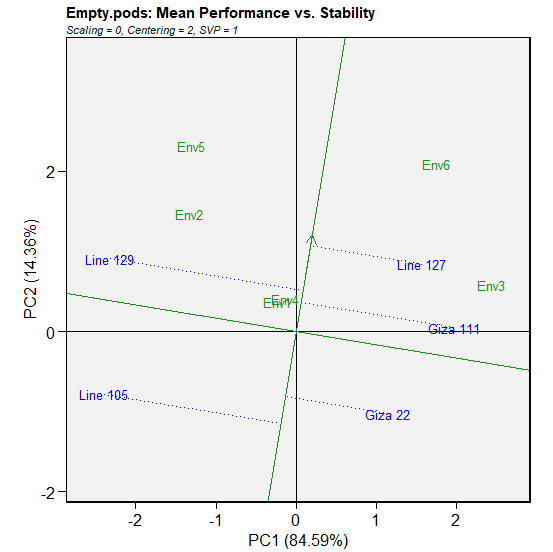


**Fig. S5** Mean performance compared with the stability biplot showing the yield-related characteristics (number of branches, seed size (mm^3^), number of pods/plant, number of empty pods) of the six settings and five genotypes of soybeans. The first two principal components (PC1 and PC2) are shown in a biplot, with PC1 representing the mean performance and PC2 indicating stability. Genotype performance and stability are determined via average environment coordination (AEC).


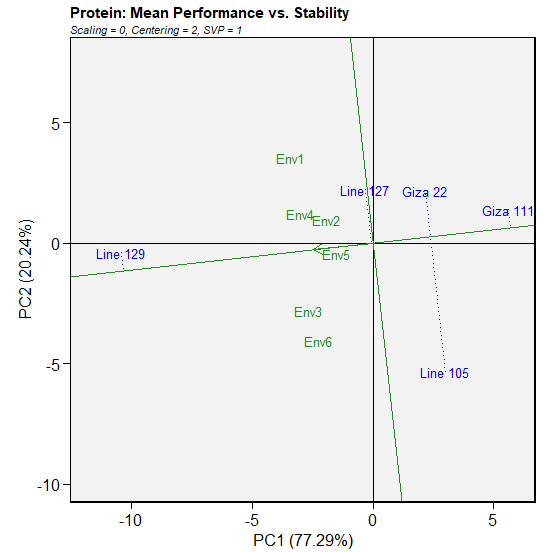

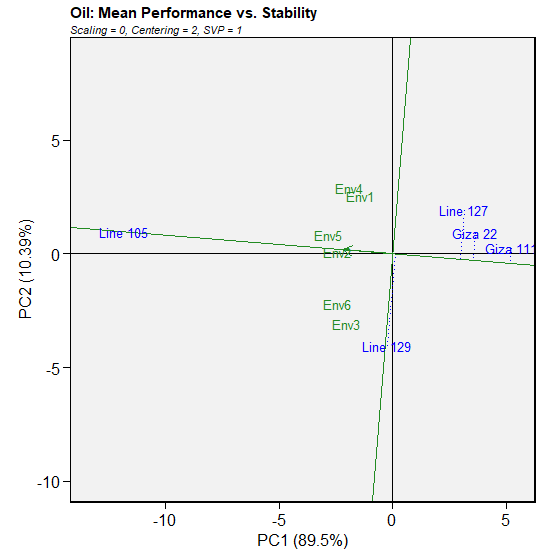


**Fig. S6** Mean performance versus consistency of protein and oil content parameters for five soybean genotypes and six environments is displayed in a biplot. PC1 and PC2, the first two primary components, are shown in a biplot. PC1 represents the mean performance, whereas PC2 represents stability. To determine genotype performance and stability, average environment coordination (or AEC) is utilized.
